# Supplementary material for: The Evolution and Characterization of the RNA Interference Pathways in Lophotrochozoa
Source: Genome Biol Evol. 2024 May 7;16(5):evae098. doi: 10.1093/gbe/evae098 (PMC11114477; doi:10.1093/gbe/evae098)
Supplement: evae098_Supplementary_Data [file evae098_supplementary_data.zip › Supplementary_materials.zip!/selected_miRNAs/NoGe_selected_miRNAs/mirdeep_pdf_files/NMRB01001187.1_Notospermus_geniculatus_strain_Ushimado_scaffold1187,_whole_genome_shotgun_sequence_26012.pdf]

Provisional ID : NMRB01001187.1_Notospermus_geniculatus_strain_Ushimado_scaffold1187,_whole_genome_shotgun_sequence_26012
Score total : 2238255.4
Score for star read(s) : 3.9
Score for read counts : 2238247.1
Score for mfe : 1.3
Score for randfold : 0
Score for cons. seed : 3
Total read count : 4390237
Mature read count : 4388416
Loop read count : 0
Star read count : 1821

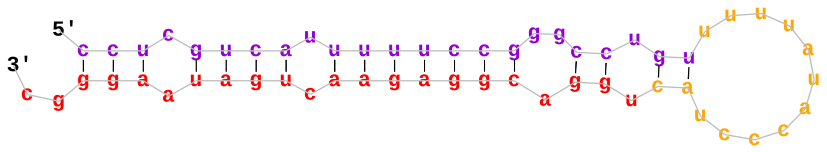

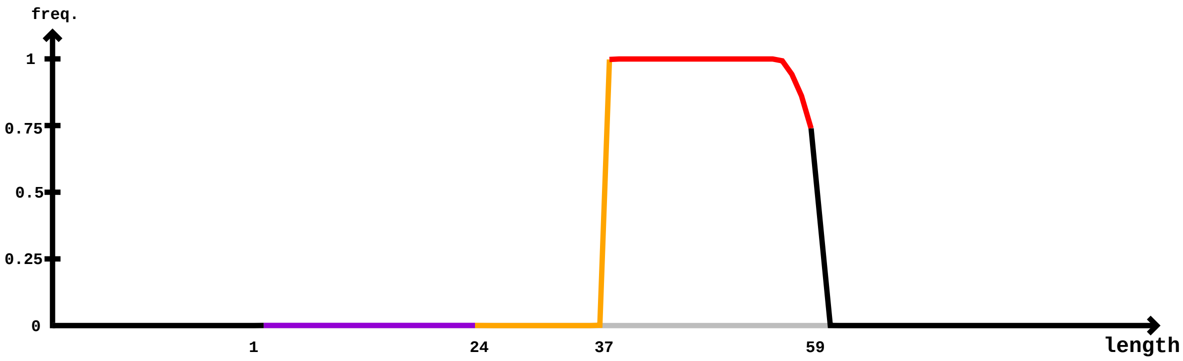

Star Mature

```
5'- gaauuuguccgaaagcgaguccucgucauuuuuccgggccuguuuuuauacccuacuggacggagaacugauaagggcucgaauaggaccaagcaacauuugacguuaucugu  -3'   obs
    gaauuuguccgaaagcgaguccucgucauuuuuccgggccuguuuuuauacccuacuggacggagaacugauaagggcucgaauaggaccaagcaacauuugacguuaucugu         exp
    ......((((.....((((((((.((((.(((((((..((.((...........)).)).))))))).)))).))))))))....))))........................    reads   mm   sample
    ..................uccucgucauuuuuccgggccugu.......................................................................    5       0    Mm2
    ..................uccAcgucauuuuuccgggccugu.......................................................................    1       1    Mm2
    ..................uccucgucauuuuuccgggccuguu......................................................................    1       0    Mm2
    ....................ccucgucauuuuuccgggccu........................................................................    2       0    Mm2
    ....................ccucgucauuuuuccgggccG........................................................................    1       1    Mm2
    ....................ccucgucauuuuuccgggccug.......................................................................    3       0    Mm2
    ....................ccucgucauuuuuccgggccugu......................................................................    338     0    Mm2
    ....................ccucgucauuuuuccgAgccugu......................................................................    1       1    Mm2
    ....................ccucgucauuuuuccggUccugu......................................................................    1       1    Mm2
    ....................ccucgucauuuuuccgggccAgu......................................................................    1       1    Mm2
    ....................ccucgucauuuuuccgUgccugu......................................................................    3       1    Mm2
    ....................ccucgucauuuuuccgggccugG......................................................................    7       1    Mm2
    ....................ccucgucauuuuuccgCgccugu......................................................................    1       1    Mm2
    ....................ccucgucauuuuuccggCccugu......................................................................    3       1    Mm2
    ....................ccucgucauuuuuccgggccuUu......................................................................    1       1    Mm2
    ....................ccucgucauuuuuccggAccugu......................................................................    2       1    Mm2
    ....................ccucgucauuuuuccgggccugC......................................................................    2       1    Mm2
    ....................Ucucgucauuuuuccgggccuguu.....................................................................    1       1    Mm2
    ....................ccucgucauuuuuccgggccuguu.....................................................................    37      0    Mm2
    ....................ccucgucauuuuuccgggccuguA.....................................................................    1       1    Mm2
    ....................ccucgucauuuuuccgggccuguuu....................................................................    10      0    Mm2
    ....................ccucgucauuuuuccgggccuguuuu...................................................................    1       0    Mm2
    ....................ccucgucauuuuuccgggccuguuuuuauacccuac.........................................................    1       0    Mm2
    ....................ccucgucauuuuuccgggccuguuuuuauacccuacuU.......................................................    1       1    Mm2
    .....................cucgucauuuuuccgggccugu......................................................................    9       0    Mm2
    .....................cucgucauuuuuccgggccuguuu....................................................................    1       0    Mm2
    .............................uuuuccgggccuguuuuuauaccc............................................................    1       0    Mm2
    ...................................................ccuacuggacggagaacugauaagggc...................................    1       0    Mm2
    ......................................................acuggacggagaacugauaag......................................    1       0    Mm2
    ......................................................acuggacggagaacugauaagg.....................................    2       0    Mm2
    ......................................................Ucuggacggagaacugauaaggg....................................    2       1    Mm2
    ......................................................Ucuggacggagaacugauaagggc...................................    8       1    Mm2
    ......................................................Ucuggacggagaacugauaagggcu..................................    3       1    Mm2
```

```
                    Star                                Mature
gaauuuguccgaaagcgaguccucgucauuuuuccgggccuguuuuuauacccuacuggacggagaacugauaagggcucgaauaggaccaagcaacauuugacguuaucugu
.......................................................cuggacggagaacugaua........................................    1     0    Mm2
.......................................................cuggacggagaacugauaa.......................................    3     0    Mm2
.......................................................cuggacggagaacugauaaU......................................    1     1    Mm2
.......................................................Uuggacggagaacugauaag......................................    1     1    Mm2
.......................................................cuggacggagaacugauaag......................................    21    0    Mm2
.......................................................cuggacggagaacugauaaA......................................    6     1    Mm2
.......................................................cuggacggagaacugauaagg.....................................    71    0    Mm2
.......................................................cuggacggagaacugauaagU.....................................    1     1    Mm2
.......................................................cuggacggagaacugauUagg.....................................    1     1    Mm2
.......................................................cuggacggagaacugauaagA.....................................    11    1    Mm2
.......................................................cuggacggagaacugaCaagg.....................................    1     1    Mm2
.......................................................cuggacggagaacugUuaaggg....................................    1     1    Mm2
.......................................................cuggacggagaacugauaaggU....................................    16    1    Mm2
.......................................................cuggacggagaacugauaaggA....................................    8     1    Mm2
.......................................................cuggGcggagaacugauaaggg....................................    1     1    Mm2
.......................................................cuggacggagaacugauaaggC....................................    1     1    Mm2
.......................................................cuggacggagaacugauaaggg....................................    97    0    Mm2
.......................................................cuggacggagaacugauaGgggc...................................    1     1    Mm2
.......................................................cAggacggagaacugauaagggc...................................    1     1    Mm2
.......................................................cuggacggagaacugauaagggc...................................    233   0    Mm2
.......................................................Uuggacggagaacugauaagggc...................................    1     1    Mm2
.......................................................cuggacggagaacugaCaagggc...................................    1     1    Mm2
.......................................................cugAacggagaacugauaagggc...................................    1     1    Mm2
.......................................................cuggacggagaacugauaagggA...................................    2     1    Mm2
.......................................................Auggacggagaacugauaagggc...................................    2     1    Mm2
.......................................................cuggacggagaacugauGagggc...................................    1     1    Mm2
.......................................................cuggacggagaacugauaagggU...................................    5     1    Mm2
.......................................................cuggacggagaacugauaagggcu..................................    18    0    Mm2
.......................................................cuggCcggagaacugauaagggcu..................................    1     1    Mm2
.......................................................cuggacggagaacugauaagggcC..................................    2     1    Mm2
........................................................ugCacggagaacugauaa.......................................    2     1    Mm2
........................................................uggacggagaacugauaC.......................................    8     1    Mm2
........................................................uggacggGgaacugauaa.......................................    1     1    Mm2
........................................................uggacgCagaacugauaa.......................................    1     1    Mm2
........................................................uggGcggagaacugauaa.......................................    1     1    Mm2
........................................................uggacggagaacugauaG.......................................    9     1    Mm2
........................................................uggacggagaacugUuaa.......................................    1     1    Mm2
........................................................uggacggagaacugauGa.......................................    2     1    Mm2
........................................................uggacggagaacugauCa.......................................    1     1    Mm2
........................................................uggacggagaacugauaU.......................................    39    1    Mm2
........................................................uggacggagaacugauaa.......................................    1306  0    Mm2
........................................................uggacggUgaacugauaa.......................................    2     1    Mm2
........................................................uggUcggagaacugauaa.......................................    2     1    Mm2
........................................................uggacggagaacugauUa.......................................    6     1    Mm2
........................................................uggacggagaGcugauaa.......................................    1     1    Mm2
........................................................uggacggagaacugaAaa.......................................    136   1    Mm2
........................................................uggacggagaacugauGag......................................    6     1    Mm2
........................................................Nggacggagaacugauaag......................................    4     1    Mm2
........................................................uggacggagaacugauaaC......................................    11    1    Mm2
........................................................uggacggagaacugaCaag......................................    1     1    Mm2
........................................................uggacggagaacugauaCg......................................    33    1    Mm2
........................................................uggacggagaacugaAaag......................................    2     1    Mm2
........................................................uggacggagaacugGuaag......................................    1     1    Mm2
........................................................uggacgCagaacugauaag......................................    1     1    Mm2
........................................................uggacggagaacCgauaag......................................    3     1    Mm2
........................................................Aggacggagaacugauaag......................................    2     1    Mm2
........................................................uggGcggagaacugauaag......................................    1     1    Mm2
........................................................uggacggagaacugauaUg......................................    26    1    Mm2
........................................................uggacggagaacugaGaag......................................    1     1    Mm2
........................................................uggacggagaacugauUag......................................    12    1    Mm2
........................................................uggacggagaacugauaaA......................................    3696  1    Mm2
........................................................uggacggagaCcugauaag......................................    10    1    Mm2
........................................................uggacggagaacugauCag......................................    40    1    Mm2
........................................................uAgacggagaacugauaag......................................    4     1    Mm2
........................................................uggacggagaGcugauaag......................................    20    1    Mm2
........................................................uggacAgagaacugauaag......................................    1     1    Mm2
........................................................uggacggagGacugauaag......................................    12    1    Mm2
........................................................uggacggUgaacugauaag......................................    1     1    Mm2
........................................................uUgacggagaacugauaag......................................    1     1    Mm2
........................................................uggacggagaacugauaag......................................    8357  0    Mm2
```

```
                    Star                            Mature
gaauuuguccgaaagcgaguccucgucauuuuuccgggccuguuuuuauacccuacuggacggagaacugauaagggcucgaauaggaccaagcaacauuugacguuaucugu
........................................................uggacUgagaacugauaag......................................      3      1      Mm2
........................................................uCgacggagaacugauaag......................................      1      1      Mm2
........................................................uggacggGgaacugauaag......................................      2      1      Mm2
........................................................uggacNgagaacugauaag......................................      1      1      Mm2
........................................................uggacggagaacugauaGg.....................................      24     1      Mm2
........................................................uggacgUagaacugauaag......................................      1      1      Mm2
........................................................uNgacggagaacugauaag......................................      1      1      Mm2
........................................................uggacggagaacuAauaag......................................      2      1      Mm2
........................................................uggacggagaacuCauaag......................................      1      1      Mm2
........................................................uggaAggagaacugauaag......................................      1      1      Mm2
........................................................uggUcggagaacugauaag......................................      1      1      Mm2
........................................................uggacggagUacugauaag......................................      2      1      Mm2
........................................................Cggacggagaacugauaag......................................      2      1      Mm2
........................................................ugAacggagaacugauaag......................................      2      1      Mm2
........................................................uggacggaUaacugauaag......................................      2      1      Mm2
........................................................uggacggagaacugUuaag......................................      1      1      Mm2
........................................................uggacggagaacugauaaU......................................      1753   1      Mm2
........................................................ugCacggagaacugauaag......................................      1      1      Mm2
........................................................uggaUggagaacugauaag......................................      1      1      Mm2
........................................................Aggacggagaacugauaagg.....................................      32     1      Mm2
........................................................uggacggagaacugauaagC.....................................      10     1      Mm2
........................................................uggacggagaacugauaCgg.....................................      71     1      Mm2
........................................................uggacNgagaacugauaagg.....................................      3      1      Mm2
........................................................ugCacggagaacugauaagg.....................................      1      1      Mm2
........................................................uggacggagaaUugauaagg.....................................      3      1      Mm2
........................................................uggacggagaacCgauaagg.....................................      2      1      Mm2
........................................................uggacgNagaacugauaagg.....................................      3      1      Mm2
........................................................ugUacggagaacugauaagg.....................................      6      1      Mm2
........................................................uggacggagaacugGuaagg.....................................      2      1      Mm2
........................................................uggacggagaacuAauaagg.....................................      1      1      Mm2
........................................................uggUcggagaacugauaagg.....................................      1      1      Mm2
........................................................uggacgUagaacugauaagg.....................................      8      1      Mm2
........................................................uggacggagaacugaCaagg.....................................      3      1      Mm2
........................................................uggaGggagaacugauaagg.....................................      1      1      Mm2
........................................................uUgacggagaacugauaagg.....................................      5      1      Mm2
........................................................uggGcggagaacugauaagg.....................................      4      1      Mm2
........................................................uggacggUgaacugauaagg.....................................      4      1      Mm2
........................................................uCgacggagaacugauaagg.....................................      5      1      Mm2
........................................................Nggacggagaacugauaagg.....................................      7      1      Mm2
........................................................uggaAggagaacugauaagg.....................................      2      1      Mm2
........................................................uggacggagaacugauaagA.....................................      2256   1      Mm2
........................................................uggacggagaacuUauaagg.....................................      1      1      Mm2
........................................................uggacggagaacugauaaAg.....................................      116    1      Mm2
........................................................uggacggagaacugauaGgg.....................................      90     1      Mm2
........................................................uggacggagaCcugauaagg.....................................      60     1      Mm2
........................................................uggacggaUaacugauaagg.....................................      4      1      Mm2
........................................................ugAacggagaacugauaagg.....................................      5      1      Mm2
........................................................uggacggagaacugauCagg.....................................      35     1      Mm2
........................................................uggacggagCacugauaagg.....................................      2      1      Mm2
........................................................uggacggagGacugauaagg.....................................      24     1      Mm2
........................................................uggacggagaacuCauaagg.....................................      4      1      Mm2
........................................................Gggacggagaacugauaagg.....................................      1      1      Mm2
........................................................uggacggagUacugauaagg.....................................      3      1      Mm2
........................................................uggacggaAaacugauaagg.....................................      1      1      Mm2
........................................................Cggacggagaacugauaagg.....................................      4      1      Mm2
........................................................uggacggagaacugauGagg.....................................      16     1      Mm2
........................................................uggacggagaacugUuaagg.....................................      1      1      Mm2
........................................................uggaUggagaacugauaagg.....................................      1      1      Mm2
........................................................uggCcggagaacugauaagg.....................................      3      1      Mm2
........................................................uggacggagaacugauaagg.....................................      18110  0      Mm2
........................................................uggacUgagaacugauaagg.....................................      11     1      Mm2
........................................................uggacggagaacugauaUgg.....................................      63     1      Mm2
........................................................uggacgAagaacugauaagg.....................................      1      1      Mm2
........................................................uggacAgagaacugauaagg.....................................      4      1      Mm2
........................................................uggacggagaacugauaaUg.....................................      1      1      Mm2
........................................................uggacggagaacugauUagg.....................................      30     1      Mm2
........................................................uggacggGgaacugauaagg.....................................      1      1      Mm2
........................................................uAgacggagaacugauaagg.....................................      13     1      Mm2
........................................................uggacggagaacugauaagU.....................................      554    1      Mm2
........................................................uggacggagaacGgauaagg.....................................      1      1      Mm2
```

Star Mature

```
gaauuuguccgaaagcgaguccucgucauuuuuccgggccuguuuuuauacccuacuggacggagaacugauaagggcucgaauaggaccaagcaacauuugacguuaucugu
........................................................uggacggagaGcugauaagg.....................................   103     1   Mm2
........................................................uggacggagaacugGuaaggg....................................   6       1   Mm2
........................................................Gggacggagaacugauaaggg....................................   1       1   Mm2
........................................................uggacggagaacuCauaaggg....................................   3       1   Mm2
........................................................uggacggagaacugauaGggg....................................   262     1   Mm2
........................................................uggacggagaacugauaUggg....................................   129     1   Mm2
........................................................uggacNgagaacugauaaggg....................................   9       1   Mm2
........................................................uggacggagaacugauaaCgg....................................   1       1   Mm2
........................................................uggCcggagaacugauaaggg....................................   3       1   Mm2
........................................................uggacggaCaacugauaaggg....................................   4       1   Mm2
........................................................uggacggagGacugauaaggg....................................   50      1   Mm2
........................................................uggacggagaacugauaagAg....................................   15      1   Mm2
........................................................uggacggagaacugauGaggg....................................   49      1   Mm2
........................................................ugAacggagaacugauaaggg....................................   14      1   Mm2
........................................................uggacgNagaacugauaaggg....................................   4       1   Mm2
........................................................uggaUggagaacugauaaggg....................................   9       1   Mm2
........................................................uggacggagaacugauaagUg....................................   6       1   Mm2
........................................................uggacgAagaacugauaaggg....................................   9       1   Mm2
........................................................uggacggagaacugauaCggg....................................   173     1   Mm2
........................................................uggacggagaacugauaagCg....................................   3       1   Mm2
........................................................uggacggagaacugauCaggg....................................   83      1   Mm2
........................................................uggacggagUacugauaaggg....................................   15      1   Mm2
........................................................uggacggaUaacugauaaggg....................................   8       1   Mm2
........................................................uggacggagaacugauUaggg....................................   62      1   Mm2
........................................................uggaAggagaacugauaaggg....................................   3       1   Mm2
........................................................uggacAgagaacugauaaggg....................................   12      1   Mm2
........................................................uggacggagCacugauaaggg....................................   3       1   Mm2
........................................................uggaGggagaacugauaaggg....................................   1       1   Mm2
........................................................uggacggaAaacugauaaggg....................................   3       1   Mm2
........................................................uAgacggagaacugauaaggg....................................   11      1   Mm2
........................................................uggacggagaacugaCaaggg....................................   4       1   Mm2
........................................................uggacggagaGcugauaaggg....................................   158     1   Mm2
........................................................uUgacggagaacugauaaggg....................................   12      1   Mm2
........................................................ugUacggagaacugauaaggg....................................   8       1   Mm2
........................................................uggacgCagaacugauaaggg....................................   5       1   Mm2
........................................................uggacggagaacuAauaaggg....................................   7       1   Mm2
........................................................uggacggUgaacugauaaggg....................................   8       1   Mm2
........................................................uggacggagaaGugauaaggg....................................   1       1   Mm2
........................................................ugCacggagaacugauaaggg....................................   4       1   Mm2
........................................................uggacggagaacugauaaAgg....................................   5       1   Mm2
........................................................Aggacggagaacugauaaggg....................................   41      1   Mm2
........................................................uggacggGgaacugauaaggg....................................   10      1   Mm2
........................................................uggacggagaacugUuaaggg....................................   6       1   Mm2
........................................................uggacUgagaacugauaaggg....................................   14      1   Mm2
........................................................uggacggagaacuUauaaggg....................................   11      1   Mm2
........................................................Nggacggagaacugauaaggg....................................   22      1   Mm2
........................................................Cggacggagaacugauaaggg....................................   8       1   Mm2
........................................................uggUcggagaacugauaaggg....................................   2       1   Mm2
........................................................uggacggagaacugauaaggg....................................   41055   0   Mm2
........................................................uggacggagaCcugauaaggg....................................   90      1   Mm2
........................................................uggacgUagaacugauaaggg....................................   10      1   Mm2
........................................................uggacggagaacCgauaaggg....................................   7       1   Mm2
........................................................uggacggagaacGgauaaggg....................................   4       1   Mm2
........................................................uggacggagaaAugauaaggg....................................   3       1   Mm2
........................................................uggacggagaacugCuaaggg....................................   5       1   Mm2
........................................................uggacggagaacugauaaggU....................................   899     1   Mm2
........................................................uggacggagaacugauaaggA....................................   5255    1   Mm2
........................................................uggacggCgaacugauaaggg....................................   4       1   Mm2
........................................................uggacCgagaacugauaaggg....................................   1       1   Mm2
........................................................uggacggagaacugauaaUgg....................................   5       1   Mm2
........................................................uggacggagaaUugauaaggg....................................   16      1   Mm2
........................................................uggacggagaacugauaaggC....................................   215     1   Mm2
........................................................uggGcggagaacugauaaggg....................................   7       1   Mm2
........................................................uggacggagaacugaAaagggc...................................   14      1   Mm2
........................................................uggacggagaaUugauaagggc...................................   79      1   Mm2
........................................................uggacggagUacugauaagggc...................................   112     1   Mm2
........................................................uggacggagaacugauaagggA...................................   11213   1   Mm2
........................................................uggacggagaacugauaaggCc...................................   101     1   Mm2
........................................................uggacggagaGcugauaagggc...................................   844     1   Mm2
........................................................uggacggagaacugauaagUgc...................................   132     1   Mm2
```

```
                    Star                                   Mature
gaauuuguccgaaagcgaguccucgucauuuuuccgggccuguuuuuauacccuacuggacggagaacugauaagggcucgaauaggaccaagcaacauuugacguuaucugu
........................................................uggUcggagaacugauaagggc...................................    89      1    Mm2
........................................................uggacggagaacugauaaUggc...................................    76      1    Mm2
........................................................uggacAgagaacugauaagggc...................................    82      1    Mm2
........................................................uggacggagaacCgauaagggc...................................    74      1    Mm2
........................................................uggaGggagaacugauaagggc...................................    18      1    Mm2
........................................................uggacggGgaacugauaagggc...................................    113     1    Mm2
........................................................uggacggaAaacugauaagggc...................................    31      1    Mm2
........................................................uggacggagaacugauaagAgc...................................    84      1    Mm2
........................................................uggacgAagaacugauaagggc...................................    63      1    Mm2
........................................................uggacggagGacugauaagggc...................................    998     1    Mm2
........................................................uggacggagaacugauUagggc...................................    638     1    Mm2
........................................................uggacgCagaacugauaagggc...................................    18      1    Mm2
........................................................uggacggagaacugUuaagggc...................................    68      1    Mm2
........................................................uggaAggagaacugauaagggc...................................    29      1    Mm2
........................................................uggacggaUaacugauaagggc...................................    93      1    Mm2
........................................................uggacgUagaacugauaagggc...................................    127     1    Mm2
........................................................uggacggagaacugauaagggU...................................    3929    1    Mm2
........................................................ugNacggagaacugauaagggc...................................    5       1    Mm2
........................................................uggacggagaacugauaaggUc...................................    123     1    Mm2
........................................................uggacNgagaacugauaagggc...................................    79      1    Mm2
........................................................uggGcggagaacugauaagggc...................................    131     1    Mm2
........................................................ugAacggagaacugauaagggc...................................    103     1    Mm2
........................................................uggacgNagaacugauaagggc...................................    22      1    Mm2
........................................................uggacggagaacugGuaagggc...................................    51      1    Mm2
........................................................uggacggagCacugauaagggc...................................    55      1    Mm2
........................................................uggacggagaacugaGaagggc...................................    8       1    Mm2
........................................................uggacCgagaacugauaagggc...................................    13      1    Mm2
........................................................uggacggagaacugauaagggc...................................    505969  0    Mm2
........................................................uggacggagaacugauaagCgc...................................    25      1    Mm2
........................................................ugUacggagaacugauaagggc...................................    92      1    Mm2
........................................................uggaUggagaacugauaagggc...................................    99      1    Mm2
........................................................uggacggagaacugauaaCggc...................................    14      1    Mm2
........................................................uggacggagaacugauaagggG...................................    327     1    Mm2
........................................................uggacggagaacuUauaagggc...................................    45      1    Mm2
........................................................uggCcggagaacugauaagggc...................................    116     1    Mm2
........................................................uggacggagaacugauaGgggc...................................    5049    1    Mm2
........................................................uggacggagaacAgauaagggc...................................    20      1    Mm2
........................................................Cggacggagaacugauaagggc...................................    64      1    Mm2
........................................................uCgacggagaacugauaagggc...................................    37      1    Mm2
........................................................Gggacggagaacugauaagggc...................................    27      1    Mm2
........................................................uggacggagaacugauaCgggc...................................    1933    1    Mm2
........................................................Nggacggagaacugauaagggc...................................    258     1    Mm2
........................................................uggacggagaacugauaUgggc...................................    1591    1    Mm2
........................................................uggacggagaCcugauaagggc...................................    435     1    Mm2
........................................................uggacUgagaacugauaagggc...................................    84      1    Mm2
........................................................uggacggagaacugauaaAggc...................................    46      1    Mm2
........................................................uggacggUgaacugauaagggc...................................    113     1    Mm2
........................................................uggacggCgaacugauaagggc...................................    23      1    Mm2
........................................................uggacggagaacugCuaagggc...................................    17      1    Mm2
........................................................uggacggagaacugauaNgggc...................................    1       1    Mm2
........................................................uggacggagaacugauaaggAc...................................    162     1    Mm2
........................................................uggacggagaaGugauaagggc...................................    14      1    Mm2
........................................................uggacggagaacuAauaagggc...................................    105     1    Mm2
........................................................uggacggagaacuCauaagggc...................................    36      1    Mm2
........................................................uggacggagaacugauGagggc...................................    745     1    Mm2
........................................................uggacggagaacGgauaagggc...................................    22      1    Mm2
........................................................uNgacggagaacugauaagggc...................................    7       1    Mm2
........................................................uggacggaCaacugauaagggc...................................    27      1    Mm2
........................................................uggacggagaacugaCaagggc...................................    45      1    Mm2
........................................................Aggacggagaacugauaagggc...................................    129     1    Mm2
........................................................uUgacggagaacugauaagggc...................................    92      1    Mm2
........................................................uggacggagaaAugauaagggc...................................    14      1    Mm2
........................................................ugCacggagaacugauaagggc...................................    46      1    Mm2
........................................................uggacggagaacugauCagggc...................................    1033    1    Mm2
........................................................uAgacggagaacugauaagggc...................................    244     1    Mm2
........................................................uggacggagaacugauaagggUu..................................    632     1    Mm2
........................................................uggacggagaaUugauaagggcu..................................    3       1    Mm2
........................................................ugCacggagaacugauaagggcu..................................    2       1    Mm2
........................................................uggacggagaacugauUagggcu..................................    27      1    Mm2
........................................................uggacggagaacugauaaggUcu..................................    8       1    Mm2
```

```
                                   Star                          Mature
gaauuuguccgaaagcgaguccucgucauuuuuccgggccuguuuuuauacccuacuggacggagaacugauaagggcucgaauaggaccaagcaacauuugacguuaucugu
........................................................uggCcggagaacugauaagggcu..................................   6      1   Mm2
........................................................uggacAgagaacugauaagggcu..................................   3      1   Mm2
........................................................uggacggagaacugauaagggAu..................................   672    1   Mm2
........................................................uggacggagaacugauaaggAcu..................................   5      1   Mm2
........................................................uggacggagaacuAauaagggcu..................................   3      1   Mm2
........................................................uggacggagaaGugauaagggcu..................................   2      1   Mm2
........................................................uggacggaCaacugauaagggcu..................................   1      1   Mm2
........................................................uggacggagaacugUuaagggcu..................................   10     1   Mm2
........................................................uggacggagaacugaCaagggcu..................................   3      1   Mm2
........................................................uggaUggagaacugauaagggcu..................................   8      1   Mm2
........................................................uggacggagUacugauaagggcu..................................   8      1   Mm2
........................................................uAgacggagaacugauaagggcu..................................   10     1   Mm2
........................................................uggacggagaacGgauaagggcu..................................   3      1   Mm2
........................................................uggacggagaacugauaaCggcu..................................   4      1   Mm2
........................................................uggacggagaacugauaagggGu..................................   10     1   Mm2
........................................................uUgacggagaacugauaagggcu..................................   7      1   Mm2
........................................................uggacggagaacugauGagggcu..................................   36     1   Mm2
........................................................Nggacggagaacugauaagggcu..................................   10     1   Mm2
........................................................uggacggGgaacugauaagggcu..................................   9      1   Mm2
........................................................uggacgNagaacugauaagggcu..................................   1      1   Mm2
........................................................uggacggagCacugauaagggcu..................................   6      1   Mm2
........................................................uggacggagGacugauaagggcu..................................   45     1   Mm2
........................................................ugUacggagaacugauaagggcu..................................   5      1   Mm2
........................................................uggacggagaacugauaGgggcu..................................   340    1   Mm2
........................................................uggaAggagaacugauaagggcu..................................   3      1   Mm2
........................................................uggacggagaacugauaCgggcu..................................   93     1   Mm2
........................................................uggacggagaacugauaaUggcu..................................   3      1   Mm2
........................................................uCgacggagaacugauaagggcu..................................   2      1   Mm2
........................................................uggacCgagaacugauaagggcu..................................   2      1   Mm2
........................................................uggaGggagaacugauaagggcu..................................   2      1   Mm2
........................................................uggacggagaacAgauaagggcu..................................   2      1   Mm2
........................................................uggacggagaacugCuaagggcu..................................   1      1   Mm2
........................................................Aggacggagaacugauaagggcu..................................   8      1   Mm2
........................................................uggacggagaacugauaagggcG..................................   1203   1   Mm2
........................................................uggacgUagaacugauaagggcu..................................   7      1   Mm2
........................................................uggGcggagaacugauaagggcu..................................   8      1   Mm2
........................................................uggacggagaacugGuaagggcu..................................   5      1   Mm2
........................................................uggacggaUaacugauaagggcu..................................   3      1   Mm2
........................................................uggacggagaacugauCagggcu..................................   47     1   Mm2
........................................................uggacUgagaacugauaagggcu..................................   5      1   Mm2
........................................................uggacgAagaacugauaagggcu..................................   4      1   Mm2
........................................................uggacggagaacugauaagggcC..................................   305    1   Mm2
........................................................uggacggagaacuUauaagggcu..................................   3      1   Mm2
........................................................uggacggagaacCgauaagggcu..................................   2      1   Mm2
........................................................uggacggagaacugauaUgggcu..................................   74     1   Mm2
........................................................uggUcggagaacugauaagggcu..................................   1      1   Mm2
........................................................uggacNgagaacugauaagggcu..................................   2      1   Mm2
........................................................uggacggagaCcugauaagggcu..................................   13     1   Mm2
........................................................Cggacggagaacugauaagggcu..................................   5      1   Mm2
........................................................uggacggagaGcugauaagggcu..................................   37     1   Mm2
........................................................uggacggagaacugauaaggCcu..................................   2      1   Mm2
........................................................uggacggagaacuCauaagggcu..................................   2      1   Mm2
........................................................uggacggUgaacugauaagggcu..................................   6      1   Mm2
........................................................ugAacggagaacugauaagggcu..................................   4      1   Mm2
........................................................uggacgCagaacugauaagggcu..................................   2      1   Mm2
........................................................uggacggaAaacugauaagggcu..................................   2      1   Mm2
........................................................uggacggagaacugauaagggcu..................................   25196  0   Mm2
........................................................uggacggagaacugauaCgggcuc.................................   1      1   Mm2
........................................................uggacggagaUcugauaagggcuc.................................   9      1   Mm2
........................................................uggacggagaacugauaagggcuc.................................   73     0   Mm2
........................................................uggacggagaacugauaagggcCc.................................   1      1   Mm2
........................................................uggacggagaacugauaagggcuA.................................   123    1   Mm2
........................................................uggacggagaacugauaGgggcuc.................................   1      1   Mm2
........................................................uggacggagaacugauaagggcAc.................................   27     1   Mm2
........................................................uggacggagaacugauaagggcuG.................................   51     1   Mm2
........................................................uggacggagaacugauaagggcuGg................................   1      1   Mm2
........................................................uggacggagaacugauaagggcucU................................   2      1   Mm2
.........................................................ggacggagaacugauaaU......................................   1      1   Mm2
.........................................................ggacggagaacugauaaA......................................   8      1   Mm2
.........................................................ggacggagaacugauaag......................................   17     0   Mm2
```

Star Mature

```
gaauuuguccgaaagcgaguccucgucauuuuuccgggccuguuuuuauacccuacuggacggagaacugauaagggcucgaauaggaccaagcaacauuugacguuaucugu
.........................................................ggacggagaaAugauaag......................................   1     1    Mm2
.........................................................ggacggagaGcugauaagg.....................................   1     1    Mm2
.........................................................Ugacggagaacugauaagg.....................................   2     1    Mm2
.........................................................ggGcggagaacugauaagg.....................................   1     1    Mm2
.........................................................ggacggagaacugauaUgg.....................................   1     1    Mm2
.........................................................ggacggagaacuUauaagg.....................................   1     1    Mm2
.........................................................ggacggagaacugauaagU.....................................   1     1    Mm2
.........................................................ggacggagaacugauaagg.....................................   47    0    Mm2
.........................................................ggacggagaacugauaagA.....................................   5     1    Mm2
.........................................................ggacggagaacugaCaaggg....................................   1     1    Mm2
.........................................................Ugacggagaacugauaaggg....................................   1     1    Mm2
.........................................................ggacggagaaAugauaaggg....................................   2     1    Mm2
.........................................................ggacggagaacugauaaggC....................................   2     1    Mm2
.........................................................ggacggagaacugauaaggA....................................   11    1    Mm2
.........................................................ggacggagaacugauaaggg....................................   48    0    Mm2
.........................................................ggacggagUacugauaaggg....................................   1     1    Mm2
.........................................................ggacggagaacugauaaggU....................................   3     1    Mm2
.........................................................ggacggaAaacugauaaggg....................................   1     1    Mm2
.........................................................ggacggagaacugauaaggAc...................................   2     1    Mm2
.........................................................ggacggagaacugauaagggA...................................   26    1    Mm2
.........................................................ggUcggagaacugauaagggc...................................   2     1    Mm2
.........................................................gAacggagaacugauaagggc...................................   2     1    Mm2
.........................................................ggaGggagaacugauaagggc...................................   1     1    Mm2
.........................................................ggacggagaacugauCagggc...................................   3     1    Mm2
.........................................................ggacggagaacugauaGgggc...................................   12    1    Mm2
.........................................................ggacggagUacugauaagggc...................................   1     1    Mm2
.........................................................ggacggagaGcugauaagggc...................................   1     1    Mm2
.........................................................ggacggaAaacugauaagggc...................................   1     1    Mm2
.........................................................ggacggagaacuUauaagggc...................................   2     1    Mm2
.........................................................ggacggagaaAugauaagggc...................................   2     1    Mm2
.........................................................ggacggagaacugauaaggCc...................................   5     1    Mm2
.........................................................ggacggagaacugauaUgggc...................................   2     1    Mm2
.........................................................ggacgUagaacugauaagggc...................................   1     1    Mm2
.........................................................ggacggagaacugauaagggU...................................   9     1    Mm2
.........................................................ggacggagaacugauaagCgc...................................   2     1    Mm2
.........................................................ggacggagaacugauaCgggc...................................   4     1    Mm2
.........................................................ggacggagaacugauaaggUc...................................   3     1    Mm2
.........................................................ggacggagaacugauaaUggc...................................   1     1    Mm2
.........................................................ggacggagCacugauaagggc...................................   8     1    Mm2
.........................................................ggacCgagaacugauaagggc...................................   1     1    Mm2
.........................................................ggacggagaacugauaagAgc...................................   1     1    Mm2
.........................................................Ugacggagaacugauaagggc...................................   20    1    Mm2
.........................................................ggacggagaCcugauaagggc...................................   1     1    Mm2
.........................................................ggacgAagaacugauaagggc...................................   1     1    Mm2
.........................................................ggacggagaacugauaagggc...................................   887   0    Mm2
.........................................................ggacggGgaacugauaagggc...................................   1     1    Mm2
.........................................................ggacggagaaUugauaagggc...................................   3     1    Mm2
.........................................................ggacggagaacugauaagUgc...................................   1     1    Mm2
.........................................................ggacggagaaGugauaagggc...................................   4     1    Mm2
.........................................................ggaAggagaacugauaagggc...................................   1     1    Mm2
.........................................................ggGcggagaacugauaagggc...................................   2     1    Mm2
.........................................................ggacggagGacugauaagggcu..................................   2     1    Mm2
.........................................................ggacCgagaacugauaagggcu..................................   1     1    Mm2
.........................................................ggacggagaacugauaagggcu..................................   33    0    Mm2
.........................................................ggacggagaacugauaagggcG..................................   3     1    Mm2
.........................................................ggacggagaacugauaaUggcu..................................   1     1    Mm2
.........................................................ggacggagaacugauaaCggcu..................................   1     1    Mm2
.........................................................ggacggagaacugauaagggcC..................................   1     1    Mm2
.........................................................Ugacggagaacugauaagggcu..................................   1     1    Mm2
.........................................................ggacggagaacugauaagggAu..................................   1     1    Mm2
.........................................................ggacggagaacugauaagggUu..................................   1     1    Mm2
.........................................................ggacggagaacugauaaggUcu..................................   1     1    Mm2
..........................................................gacggagaacugauaagggc...................................   1     0    Mm2
...........................................................acggagaacugauaaggg....................................   1     0    Mm2
...........................................................acUgagaacugauaagggc...................................   1     1    Mm2
...........................................................acggagaacugauaagggc...................................   3     0    Mm2
............................................................Uggagaacugauaagggc...................................   3     1    Mm2
............................................................cggagaacugauaagggc...................................   1     0    Mm2

....................ccucgucauuuuuccgggccugu......................................................................   13    0    Mm1
```

```
                    Star                      Mature
gaauuuguccgaaagcgaguccucgucauuuuuccgggccuguuuuuauacccuacuggacggagaacugauaagggcucgaauaggaccaagcaacauuugacguuaucugu
....................ccucgucauuuuuccgUgccugu......................................................................	1	1	Mm1
.....................................................Ucuggacggagaacugauaagggc....................................	1	1	Mm1
......................................................cuggacggagaacugauaag.......................................	1	0	Mm1
......................................................cuggacggagaacugauaagg......................................	43	0	Mm1
......................................................cuggacggagaacugauaagA......................................	7	1	Mm1
......................................................cuggacggagCacugauaagg......................................	1	1	Mm1
......................................................cuggacggagCacugauaaggg.....................................	1	1	Mm1
......................................................cuggacggagaacugauaaggg.....................................	17	0	Mm1
......................................................cuggacggagaacugauaagggc....................................	45	0	Mm1
......................................................cuggacggagCacugauaagggc....................................	1	1	Mm1
......................................................cugUacggagaacugauaagggc....................................	1	1	Mm1
........................................................uggacggagaacugaAaa.......................................	64	1	Mm1
........................................................uggacggagaacugauaU.......................................	29	1	Mm1
........................................................ugAacggagaacugauaa.......................................	1	1	Mm1
........................................................uggacUgagaacugauaa.......................................	1	1	Mm1
........................................................uggacggagaacugauaC.......................................	2	1	Mm1
........................................................uggacggagaacugauaG.......................................	2	1	Mm1
........................................................uggacggagaacugauGa.......................................	1	1	Mm1
........................................................uAgacggagaacugauaa.......................................	2	1	Mm1
........................................................uggacgAagaacugauaa.......................................	1	1	Mm1
........................................................Nggacggagaacugauaa.......................................	2	1	Mm1
........................................................uggacggagaacugauaa.......................................	995	0	Mm1
........................................................uggacgUagaacugauaa.......................................	1	1	Mm1
........................................................uggacggagaacuAauaa.......................................	1	1	Mm1
........................................................Aggacggagaacugauaa.......................................	1	1	Mm1
........................................................uggacggagaGcugauaa.......................................	1	1	Mm1
........................................................uggacggagaacugauUa.......................................	6	1	Mm1
........................................................uggacggagaacugauCa.......................................	3	1	Mm1
........................................................uggacggagaacugaCaa.......................................	1	1	Mm1
........................................................uggacggagaacugauaUg......................................	14	1	Mm1
........................................................uggacggagaCcugauaag......................................	7	1	Mm1
........................................................uggacggagaGcugauaag......................................	19	1	Mm1
........................................................uggaUggagaacugauaag......................................	1	1	Mm1
........................................................uggUcggagaacugauaag......................................	1	1	Mm1
........................................................uggacggagUacugauaag......................................	2	1	Mm1
........................................................uggacggagaacugauUag......................................	5	1	Mm1
........................................................uggacggagaacugauaGg......................................	15	1	Mm1
........................................................uAgacggagaacugauaag......................................	2	1	Mm1
........................................................uggacgCagaacugauaag......................................	1	1	Mm1
........................................................uggacggaAaacugauaag......................................	2	1	Mm1
........................................................uggacAgagaacugauaag......................................	2	1	Mm1
........................................................uggacggagGacugauaag......................................	6	1	Mm1
........................................................uggacggagaacugauGag......................................	3	1	Mm1
........................................................uggacggagaacugauaag......................................	3971	0	Mm1
........................................................uggacggagaacugauaaA......................................	2940	1	Mm1
........................................................Nggacggagaacugauaag......................................	3	1	Mm1
........................................................uggacggagaacugUuaag......................................	2	1	Mm1
........................................................Aggacggagaacugauaag......................................	3	1	Mm1
........................................................uggacggagaacugauaCg......................................	16	1	Mm1
........................................................ugAacggagaacugauaag......................................	1	1	Mm1
........................................................uggacggagaacugauaaU......................................	1409	1	Mm1
........................................................uggacggagaacugGuaag......................................	1	1	Mm1
........................................................ugCacggagaacugauaag......................................	1	1	Mm1
........................................................uggacgAagaacugauaag......................................	1	1	Mm1
........................................................uggacggagaacugauCag......................................	2	1	Mm1
........................................................uggacggagaacugauaaC......................................	20	1	Mm1
........................................................uggacggagaaGugauaagg.....................................	1	1	Mm1
........................................................uggacggUgaacugauaagg.....................................	6	1	Mm1
........................................................uggacggagaacugauCagg.....................................	29	1	Mm1
........................................................uggacUgagaacugauaagg.....................................	3	1	Mm1
........................................................uggaUggagaacugauaagg.....................................	3	1	Mm1
........................................................ugAacggagaacugauaagg.....................................	1	1	Mm1
........................................................uggacggagaacugauaagU.....................................	365	1	Mm1
........................................................ugCacggagaacugauaagg.....................................	2	1	Mm1
........................................................uggacggGgaacugauaagg.....................................	1	1	Mm1
........................................................ugUacggagaacugauaagg.....................................	3	1	Mm1
........................................................Nggacggagaacugauaagg.....................................	6	1	Mm1
........................................................uggacggagaacugauaCgg.....................................	71	1	Mm1
........................................................uggacggagaCcugauaagg.....................................	70	1	Mm1
........................................................uggacggagaGcugauaagg.....................................	122	1	Mm1
```

```
                    Star                                  Mature
gaauuuguccgaaagcgaguccucgucauuuuuccgggccuguuuuuauacccuacuggacggagaacugauaagggcucgaauaggaccaagcaacauuugacguuaucugu
........................................................uAgacggagaacugauaagg.....................................    5      1    Mm1
........................................................uggacggagaacugauUagg.....................................    29     1    Mm1
........................................................uggacCgagaacugauaagg.....................................    2      1    Mm1
........................................................uggacggagaacugaAaagg.....................................    1      1    Mm1
........................................................uggacgCagaacugauaagg.....................................    1      1    Mm1
........................................................uggacggagaaUugauaagg.....................................    14     1    Mm1
........................................................uggacggagaacugauaagg.....................................    17362  0    Mm1
........................................................uggaAggagaacugauaagg.....................................    2      1    Mm1
........................................................uggGcggagaacugauaagg.....................................    4      1    Mm1
........................................................uggacggagaacAgauaagg.....................................    2      1    Mm1
........................................................uCgacggagaacugauaagg.....................................    2      1    Mm1
........................................................uggacggCgaacugauaagg.....................................    1      1    Mm1
........................................................uggacggagCacugauaagg.....................................    5      1    Mm1
........................................................uggacggagaacugauGagg.....................................    40     1    Mm1
........................................................uggacggagaacugGuaagg.....................................    3      1    Mm1
........................................................uggacggagaacugUuaagg.....................................    2      1    Mm1
........................................................uggacggagaacugaGaagg.....................................    2      1    Mm1
........................................................uggacggaUaacugauaagg.....................................    1      1    Mm1
........................................................Cggacggagaacugauaagg.....................................    3      1    Mm1
........................................................uggacggagaacugauaagC.....................................    3      1    Mm1
........................................................uggacggagaacuCauaagg.....................................    2      1    Mm1
........................................................uggacggagaacugaCaagg.....................................    1      1    Mm1
........................................................uggacgAagaacugauaagg.....................................    1      1    Mm1
........................................................uggacggagaacugauaaAg.....................................    24     1    Mm1
........................................................uggacggagaacugauaagA.....................................    1788   1    Mm1
........................................................uggacgUagaacugauaagg.....................................    7      1    Mm1
........................................................uggacNgagaacugauaagg.....................................    3      1    Mm1
........................................................Aggacggagaacugauaagg.....................................    53     1    Mm1
........................................................uggacggagaacCgauaagg.....................................    4      1    Mm1
........................................................uggacggagaacugauaGgg.....................................    77     1    Mm1
........................................................uggacggagaacugauaUgg.....................................    70     1    Mm1
........................................................uggacggagaacugauaaUg.....................................    1      1    Mm1
........................................................uggacggagUacugauaagg.....................................    6      1    Mm1
........................................................uggacAgagaacugauaagg.....................................    5      1    Mm1
........................................................uggacggagGacugauaagg.....................................    8      1    Mm1
........................................................uggaGggagaacugauaagg.....................................    1      1    Mm1
........................................................uUgacggagaacugauaagg.....................................    5      1    Mm1
........................................................uggUcggagaacugauaagg.....................................    6      1    Mm1
........................................................uggacggagaacuUauaagg.....................................    1      1    Mm1
........................................................ugAacggagaacugauaaggg....................................    3      1    Mm1
........................................................uggacggagaacugUuaaggg....................................    4      1    Mm1
........................................................uggacggagaacCgauaaggg....................................    2      1    Mm1
........................................................uggacggagUacugauaaggg....................................    7      1    Mm1
........................................................uggacggagaacugauaagCg....................................    1      1    Mm1
........................................................uggacggagGacugauaaggg....................................    3      1    Mm1
........................................................uggacggagaacuAauaaggg....................................    3      1    Mm1
........................................................uggacggagaacugGuaaggg....................................    5      1    Mm1
........................................................uggacggagaacAgauaaggg....................................    1      1    Mm1
........................................................uUgacggagaacugauaaggg....................................    1      1    Mm1
........................................................uggacgCagaacugauaaggg....................................    1      1    Mm1
........................................................ugUacggagaacugauaaggg....................................    1      1    Mm1
........................................................uggacggGgaacugauaaggg....................................    2      1    Mm1
........................................................uggacggagaCcugauaaggg....................................    33     1    Mm1
........................................................uggGcggagaacugauaaggg....................................    3      1    Mm1
........................................................uggaUggagaacugauaaggg....................................    1      1    Mm1
........................................................uggUcggagaacugauaaggg....................................    3      1    Mm1
........................................................uggacggagaacugauaaggg....................................    14223  0    Mm1
........................................................uggacggagaacugauaGggg....................................    82     1    Mm1
........................................................uAgacggagaacugauaaggg....................................    22     1    Mm1
........................................................uggacggUgaacugauaaggg....................................    4      1    Mm1
........................................................uggacggagaacugauaaUgg....................................    3      1    Mm1
........................................................uggacggagaacugauaUggg....................................    41     1    Mm1
........................................................uggacggagaaUugauaaggg....................................    1      1    Mm1
........................................................uggacggagCacugauaaggg....................................    2      1    Mm1
........................................................Aggacggagaacugauaaggg....................................    31     1    Mm1
........................................................uggacAgagaacugauaaggg....................................    3      1    Mm1
........................................................uggacggaUaacugauaaggg....................................    1      1    Mm1
........................................................uggacggagaacugauaaggC....................................    5      1    Mm1
........................................................uggacUgagaacugauaaggg....................................    3      1    Mm1
........................................................uggacggagaacugauGaggg....................................    18     1    Mm1
```

Star **Mature**

gaauuuguccgaaagcgaguccucgucauuuuuccgggccuguuuuuauacccuacuggacggagaacugauaagggcucgaauaggaccaagcaacauuugacguuaucugu			
....uggacggagaacugauaaggU....................................	413	1	Mm1
....uCgacggagaacugauaaggg....................................	3	1	Mm1
....uggacNgagaacugauaaggg....................................	1	1	Mm1
....Nggacggagaacugauaaggg....................................	6	1	Mm1
....uggacggagaacugauaagUg....................................	3	1	Mm1
....Cggacggagaacugauaaggg....................................	2	1	Mm1
....uggacggagaacugauaaggA....................................	3716	1	Mm1
....uggacggagaacugauUaggg....................................	24	1	Mm1
....uggacggagaacugauCaggg....................................	29	1	Mm1
....uggacggagaacugauaCggg....................................	59	1	Mm1
....uggacggagaGcugauaaggg....................................	85	1	Mm1
....uggacggCgaacugauaaggg....................................	1	1	Mm1
....uggacgUagaacugauaaggg....................................	2	1	Mm1
....uggacggagaaAugauaaggg....................................	1	1	Mm1
....uggacggagaacugauaCgggc...................................	394	1	Mm1
....ugAacggagaacugauaagggc...................................	19	1	Mm1
....uggacggaUaacugauaagggc...................................	19	1	Mm1
....uggacgAagaacugauaagggc...................................	8	1	Mm1
....uggacggUgaacugauaagggc...................................	47	1	Mm1
....uggacggagaacugauUagggc...................................	148	1	Mm1
....uggacggaCaacugauaagggc...................................	6	1	Mm1
....uggaAggagaacugauaagggc...................................	8	1	Mm1
....uggacggagaacugauaGgggc...................................	762	1	Mm1
....uggacggagUacugauaagggc...................................	49	1	Mm1
....uggacAgagaacugauaagggc...................................	10	1	Mm1
....uggacggagaaAugauaagggc...................................	6	1	Mm1
....uggacggagaacuUauaagggc...................................	8	1	Mm1
....uggacggagaacugauaaggUc...................................	26	1	Mm1
....uggacggagaacugaCaagggc...................................	11	1	Mm1
....uggacggagaacugauaagggG...................................	16	1	Mm1
....uggacggagaacugauGagggc...................................	154	1	Mm1
....uAgacggagaacugauaagggc...................................	47	1	Mm1
....uggacggagaacugauaagggU...................................	821	1	Mm1
....uggacggagCacugauaagggc...................................	27	1	Mm1
....uggacNgagaacugauaagggc...................................	11	1	Mm1
....uggacggagaacugUuaagggc...................................	9	1	Mm1
....uggacggCgaacugauaagggc...................................	3	1	Mm1
....uggacggagaacugauNagggc...................................	1	1	Mm1
....uggacggagaacugauaaggAc...................................	18	1	Mm1
....uggacggagaCcugauaagggc...................................	88	1	Mm1
....uggacgNagaacugauaagggc...................................	3	1	Mm1
....uCgacggagaacugauaagggc...................................	10	1	Mm1
....uggacggagaacCgauaagggc...................................	10	1	Mm1
....uggacUgagaacugauaagggc...................................	17	1	Mm1
....ugUacggagaacugauaagggc...................................	10	1	Mm1
....uNgacggagaacugauaagggc...................................	1	1	Mm1
....Nggacggagaacugauaagggc...................................	48	1	Mm1
....uggacggagaacugauaagUgc...................................	36	1	Mm1
....uggacggagaacugauaagCgc...................................	8	1	Mm1
....uggacggagaacAgauaagggc...................................	5	1	Mm1
....uggacggagaacugauCagggc...................................	209	1	Mm1
....uggacggagaacuCauaagggc...................................	12	1	Mm1
....uggaUggagaacugauaagggc...................................	20	1	Mm1
....uggacggagGacugauaagggc...................................	251	1	Mm1
....uggacggagaaGugauaagggc...................................	2	1	Mm1
....uggacggGgaacugauaagggc...................................	32	1	Mm1
....uggacggagaaUugauaagggc...................................	20	1	Mm1
....uggacggagaacugauaagggc...................................	99594	0	Mm1
....uggaGggagaacugauaagggc...................................	1	1	Mm1
....Cggacggagaacugauaagggc...................................	9	1	Mm1
....uggacggagaacugaAaagggc...................................	3	1	Mm1
....uggacggagaacGgauaagggc...................................	3	1	Mm1
....uggacggagaacugauaagggA...................................	4855	1	Mm1
....uggacggagaGcugauaagggc...................................	209	1	Mm1
....uggacggagaacugauaaCggc...................................	2	1	Mm1
....uggGcggagaacugauaagggc...................................	25	1	Mm1
....uggacggagaacugauaUgggc...................................	321	1	Mm1
....uggacggagaacugauaaggCc...................................	6	1	Mm1
....uggacgCagaacugauaagggc...................................	7	1	Mm1
....uggacggagaacugGuaagggc...................................	9	1	Mm1

Star Mature

```
gaauuuguccgaaagcgaguccucgucauuuuuccgggccuguuuuuauacccuacuggacggagaacugauaagggcucgaauaggaccaagcaacauuugacguuaucugu
........................................................uggacggagaacugauaaUggc...................................   17   1   Mm1
........................................................uggCcggagaacugauaagggc...................................   24   1   Mm1
........................................................ugCacggagaacugauaagggc...................................   8    1   Mm1
........................................................uggUcggagaacugauaagggc...................................   13   1   Mm1
........................................................uUgacggagaacugauaagggc...................................   18   1   Mm1
........................................................uggacggaAaacugauaagggc...................................   6    1   Mm1
........................................................uggacggagaacugauaagAgc...................................   24   1   Mm1
........................................................Aggacggagaacugauaagggc...................................   53   1   Mm1
........................................................uggacggagaacugCuaagggc...................................   3    1   Mm1
........................................................uggacgUagaacugauaagggc...................................   17   1   Mm1
........................................................uggacggagaacuAauaagggc...................................   18   1   Mm1
........................................................uggacggagaacugauaaAggc...................................   21   1   Mm1
........................................................uggacggagaacuAauaagggcu..................................   1    1   Mm1
........................................................uggacggagaaGugauaagggcu..................................   1    1   Mm1
........................................................uggaUggagaacugauaagggcu..................................   1    1   Mm1
........................................................uggUcggagaacugauaagggcu..................................   1    1   Mm1
........................................................uggacggagaacugauaGgggcu..................................   26   1   Mm1
........................................................uggacggagaacugauaCgggcu..................................   21   1   Mm1
........................................................uggacAgagaacugauaagggcu..................................   1    1   Mm1
........................................................uggacggagaacugauaagggUu..................................   147  1   Mm1
........................................................uAgacggagaacugauaagggcu..................................   2    1   Mm1
........................................................uggacggagaacugauaaggUcu..................................   3    1   Mm1
........................................................uggacggagaacugauaaUggcu..................................   1    1   Mm1
........................................................uggacggagaacugaCaagggcu..................................   1    1   Mm1
........................................................uggacggagaacugauaagUgcu..................................   1    1   Mm1
........................................................uggacggUgaacugauaagggcu..................................   1    1   Mm1
........................................................uggacggagaacugauaagggcu..................................   4894 0   Mm1
........................................................uggacggagaacugauaagggcC..................................   44   1   Mm1
........................................................uggacggagaacugauUagggcu..................................   11   1   Mm1
........................................................uggacggagaacugGuaagggcu..................................   2    1   Mm1
........................................................uggacggagaacugauaagCgcu..................................   1    1   Mm1
........................................................uggacggagaacugauaagggcG..................................   66   1   Mm1
........................................................uggacggagaCcugauaagggcu..................................   9    1   Mm1
........................................................uggCcggagaacugauaagggcu..................................   1    1   Mm1
........................................................uggacggagaacugauaagggGu..................................   10   1   Mm1
........................................................uggacggagaGcugauaagggcu..................................   14   1   Mm1
........................................................Aggacggagaacugauaagggcu..................................   2    1   Mm1
........................................................uggacgUagaacugauaagggcu..................................   3    1   Mm1
........................................................uggacggagaacugauaaAggcu..................................   1    1   Mm1
........................................................uggacUgagaacugauaagggcu..................................   1    1   Mm1
........................................................uggacggagUacugauaagggcu..................................   3    1   Mm1
........................................................uggacNgagaacugauaagggcu..................................   2    1   Mm1
........................................................uggacggagaacugauaUgggcu..................................   23   1   Mm1
........................................................uggacggagGacugauaagggcu..................................   14   1   Mm1
........................................................uUgacggagaacugauaagggcu..................................   2    1   Mm1
........................................................uggacggagaacugauGagggcu..................................   4    1   Mm1
........................................................uggacggaUaacugauaagggcu..................................   1    1   Mm1
........................................................ugCacggagaacugauaagggcu..................................   2    1   Mm1
........................................................uggacggagaacugUuaagggcu..................................   3    1   Mm1
........................................................uggacggagaacugauaagggAu..................................   284  1   Mm1
........................................................uggacggagaacugauCagggcu..................................   12   1   Mm1
........................................................uggacggagaacugauaagggcuc.................................   22   0   Mm1
........................................................uggacggagaacugauaagggcuA.................................   18   1   Mm1
........................................................uggacggagaacugauaagggcAc.................................   14   1   Mm1
........................................................uggacggagaUcugauaagggcuc.................................   5    1   Mm1
........................................................uggacggagaacugauaagggcuGgaa..............................   1    1   Mm1
.........................................................ggacggagaacugauaaA......................................   9    1   Mm1
.........................................................ggacggagaacugauaag......................................   9    0   Mm1
.........................................................ggacggagaaAugauaag......................................   1    1   Mm1
.........................................................ggacggagCacugauaagg.....................................   1    1   Mm1
.........................................................ggacggagaacugauaagg.....................................   27   0   Mm1
.........................................................ggacggagaacugauaagU.....................................   5    1   Mm1
.........................................................ggacggagaacugauaaggg....................................   43   0   Mm1
.........................................................Ugacggagaacugauaaggg....................................   1    1   Mm1
.........................................................ggGcggagaacugauaaggg....................................   1    1   Mm1
.........................................................ggacggagaacugauaaggC....................................   1    1   Mm1
.........................................................ggacggagaacugauaaggA....................................   7    1   Mm1
.........................................................ggacggagaCcugauaagggc...................................   1    1   Mm1
.........................................................ggacggagaacugauaagggA...................................   7    1   Mm1
.........................................................ggGcggagaacugauaagggc...................................   2    1   Mm1
```

Star **Mature**

```
gaauuuguccgaaagcgaguccucgucauuuuuccgggccuguuuuuauacccuacuggacggagaacugauaagggcucgaauaggaccaagcaacauuugacguuaucugu
.........................................................ggacggagCacugauaagggc...................................  1    1  Mm1
.........................................................ggacggagaacugauaagggc...................................  178  0  Mm1
.........................................................ggacggagGacugauaagggc...................................  2    1  Mm1
.........................................................Ugacggagaacugauaagggc...................................  4    1  Mm1
.........................................................ggacggaAaacugauaagggc...................................  1    1  Mm1
.........................................................ggaAggagaacugauaagggc...................................  1    1  Mm1
.........................................................ggacggagaacugauaagggU...................................  2    1  Mm1
.........................................................ggacggagaacGgauaagggc...................................  1    1  Mm1
.........................................................ggacggagaaAugauaagggc...................................  1    1  Mm1
.........................................................ggacggagaaGugauaagggc...................................  2    1  Mm1
.........................................................ggacggagaacugauaaggAc...................................  1    1  Mm1
.........................................................ggacggagaacugauaaUggc...................................  1    1  Mm1
.........................................................ggacggagaacugauaagggcu..................................  8    0  Mm1
............................................................Uggagaacugauaagggc...................................  1    1  Mm1

....................Ncucgucauuuuuccgggccugu......................................................................  1    1  Fm2
....................ccucgucauuuuuUcgggccugu......................................................................  1    1  Fm2
....................ccucgucauuuuuccggAccugu......................................................................  1    1  Fm2
....................ccucgucauuAuuccgggccugu......................................................................  1    1  Fm2
....................ccucgucauuuuuccgggccugu......................................................................  212  0  Fm2
....................Ucucgucauuuuuccgggccugu......................................................................  5    1  Fm2
....................ccucgucauuuuuccggUccugu......................................................................  1    1  Fm2
....................ccucgucauuuuuccgggccugG......................................................................  5    1  Fm2
....................Ucucgucauuuuuccgggccuguu.....................................................................  1    1  Fm2
....................ccucgucauuuuuccgggccuguu.....................................................................  55   0  Fm2
....................ccucgucauuuuuccgggccuguG.....................................................................  1    1  Fm2
....................ccucgucauuuuuccggUccuguuu....................................................................  1    1  Fm2
....................ccucgucauuuuuccgggccuguuu....................................................................  39   0  Fm2
....................ccucgucauuAuuccgggccuguuuu...................................................................  1    1  Fm2
....................ccucgucauuuuuccgggccugGuuu...................................................................  1    1  Fm2
....................ccucgucauuuuuccgggccuguuuu...................................................................  19   0  Fm2
....................ccucgucauuuuuccgggccuguuuuuauacccuacu........................................................  5    0  Fm2
.....................cucgucauuuuuccgggAcugu......................................................................  1    1  Fm2
.....................cucgucauuuuuccgggccugu......................................................................  12   0  Fm2
.....................cucgucauuuuuccgggccuguu.....................................................................  18   0  Fm2
...........................................uuuuauacccuacuggacggagaac.............................................  21   0  Fm2
.....................................................uacuggacggagaacugauaagggc...................................  11   0  Fm2
......................................................acuggacggagaacugaGaagg.....................................  1    1  Fm2
......................................................acuggacggagaacugauaagg.....................................  13   0  Fm2
.......................................................cuggacggagaacugaua........................................  9    0  Fm2
.......................................................cuggacggagaacugauU........................................  2    1  Fm2
.......................................................cuggacggagaacugauaa.......................................  17   0  Fm2
.......................................................cuggacggagaacugauaag......................................  51   0  Fm2
.......................................................cuggacggagaacugauCag......................................  1    1  Fm2
.......................................................cuggacggagaacugaCaag......................................  2    1  Fm2
.......................................................cuggacggagaacugauaaA......................................  3    1  Fm2
.......................................................cuggacggagaacugauaaU......................................  11   1  Fm2
.......................................................cuggacggagaacugauaagA.....................................  6    1  Fm2
.......................................................cuggacggagaacugaGaagg.....................................  4    1  Fm2
.......................................................cuggacggagaacugauGagg.....................................  2    1  Fm2
.......................................................cuAgacggagaacugauaagg.....................................  1    1  Fm2
.......................................................cuggacggagGacugauaagg.....................................  1    1  Fm2
.......................................................cuggacggagCacugauaagg.....................................  1    1  Fm2
.......................................................cuggacggagaacugauaagg.....................................  228  0  Fm2
.......................................................cuggacggagaacugauaaAg.....................................  6    1  Fm2
.......................................................cuggacggGgaacugauaagg.....................................  1    1  Fm2
.......................................................cuggacggagaacugauaagU.....................................  3    1  Fm2
.......................................................Uuggacggagaacugauaaggg....................................  1    1  Fm2
.......................................................cuggacggagaacugauaaggU....................................  45   1  Fm2
.......................................................cuggacggagaacugauaagAg....................................  1    1  Fm2
.......................................................cuggacggagaacugauaaAgg....................................  7    1  Fm2
.......................................................cuggacggagaacugauaaggA....................................  31   1  Fm2
.......................................................cuggacggagaacugaGaaggg....................................  1    1  Fm2
.......................................................cuggacggagaacugauaaggg....................................  210  0  Fm2
.......................................................cuggacggagaacugaCaaggg....................................  1    1  Fm2
.......................................................cuggacggagaacugauCaggg....................................  2    1  Fm2
.......................................................cuggacggagaacugauaagggG...................................  2    1  Fm2
.......................................................cuggacggagCacugauaagggc...................................  1    1  Fm2
.......................................................cuggacggagaacugauUagggc...................................  1    1  Fm2
.......................................................cuggacggagaacugauCagggc...................................  2    1  Fm2
```

Star Mature

```
gaauuuguccgaaagcgaguccucgucauuuuuccgggccuguuuuuauacccuacuggacggagaacugauaagggcucgaauaggaccaagcaacauuugacguuaucugu
......................................................cuggacggagaaUugauaagggc....................................   4     1   Fm2
......................................................cuggacggagaacugaCaagggc....................................   1     1   Fm2
......................................................cuggacggagaacugauaagggc....................................   420   0   Fm2
......................................................cuggacggagaacugauaaUggc....................................   1     1   Fm2
......................................................cuggacggagaacugaAaagggc....................................   2     1   Fm2
......................................................cuggacggagaacugaGaagggc....................................   2     1   Fm2
......................................................cuggacggagaacCgauaagggc....................................   1     1   Fm2
......................................................cuggacggagaacugauaagggU....................................   23    1   Fm2
......................................................cuggaUggagaacugauaagggc....................................   2     1   Fm2
......................................................cuggacggagaacugauaagggcu...................................   6     0   Fm2
........................................................uggacggagaacugauUa.......................................   9     1   Fm2
........................................................uggacggagaacugauaa.......................................   908   0   Fm2
........................................................uggacggagaGcugauaa.......................................   1     1   Fm2
........................................................uggacggagaacugaAaa.......................................   81    1   Fm2
........................................................uggacggagaacugauaU.......................................   23    1   Fm2
........................................................uggacggagaacugauaC.......................................   3     1   Fm2
........................................................uggacggagaacugauGa.......................................   3     1   Fm2
........................................................uggacggagaacugaGaa.......................................   2     1   Fm2
........................................................uggacggagaCcugauaa.......................................   2     1   Fm2
........................................................uggacggagaacugauCa.......................................   1     1   Fm2
........................................................uggacggaUaacugauaa.......................................   1     1   Fm2
........................................................uggaUggagaacugauaa.......................................   1     1   Fm2
........................................................uggacggagaacugauaG.......................................   12    1   Fm2
........................................................ugAacggagaacugauaag......................................   3     1   Fm2
........................................................uggacggagaacAgauaag......................................   1     1   Fm2
........................................................uAgacggagaacugauaag......................................   6     1   Fm2
........................................................uggacggagaacugauGag......................................   9     1   Fm2
........................................................uggacggagaacugauaaU......................................   1662  1   Fm2
........................................................Cggacggagaacugauaag......................................   1     1   Fm2
........................................................uggacggagaacugauaaA......................................   3153  1   Fm2
........................................................uggacggaUaacugauaag......................................   1     1   Fm2
........................................................uggacggagaacuCauaag......................................   2     1   Fm2
........................................................uUgacggagaacugauaag......................................   1     1   Fm2
........................................................uggacggagaacugauaCg......................................   22    1   Fm2
........................................................uggCcggagaacugauaag......................................   1     1   Fm2
........................................................uggacggagaacugaAaag......................................   5     1   Fm2
........................................................uggaUggagaacugauaag......................................   9     1   Fm2
........................................................uggacggagaacCgauaag......................................   1     1   Fm2
........................................................uggacggagaGcugauaag......................................   18    1   Fm2
........................................................uggaGggagaacugauaag......................................   2     1   Fm2
........................................................uggacggagCacugauaag......................................   2     1   Fm2
........................................................ugCacggagaacugauaag......................................   1     1   Fm2
........................................................uggacggagaacugaCaag......................................   3     1   Fm2
........................................................uggacggUgaacugauaag......................................   2     1   Fm2
........................................................Nggacggagaacugauaag......................................   3     1   Fm2
........................................................uggacggGgaacugauaag......................................   3     1   Fm2
........................................................Aggacggagaacugauaag......................................   1     1   Fm2
........................................................uggacNgagaacugauaag......................................   2     1   Fm2
........................................................uggacggagaacugauUag......................................   9     1   Fm2
........................................................uggUcggagaacugauaag......................................   1     1   Fm2
........................................................uggacggCgaacugauaag......................................   1     1   Fm2
........................................................uggacggagGacugauaag......................................   9     1   Fm2
........................................................uggacUgagaacugauaag......................................   2     1   Fm2
........................................................uggacAgagaacugauaag......................................   1     1   Fm2
........................................................uggGcggagaacugauaag......................................   1     1   Fm2
........................................................uggacggagaaUugauaag......................................   2     1   Fm2
........................................................uggacggagUacugauaag......................................   4     1   Fm2
........................................................uggacggagaacugauaGg......................................   19    1   Fm2
........................................................uggacggagaCcugauaag......................................   8     1   Fm2
........................................................uggacgUagaacugauaag......................................   2     1   Fm2
........................................................uggacggagaacugauaUg......................................   17    1   Fm2
........................................................uggacggagaacugauaaC......................................   7     1   Fm2
........................................................uggacggagaacugauCag......................................   15    1   Fm2
........................................................uggacggagaacugauaag......................................   7644  0   Fm2
........................................................ugUacggagaacugauaagg.....................................   4     1   Fm2
........................................................uggacggagaacugauaUgg.....................................   70    1   Fm2
........................................................uggGcggagaacugauaagg.....................................   3     1   Fm2
........................................................uUgacggagaacugauaagg.....................................   9     1   Fm2
........................................................uggacggagaacugauCagg.....................................   47    1   Fm2
........................................................uggacCgagaacugauaagg.....................................   1     1   Fm2
```

```
                    Star                                  Mature
gaauuuguccgaaagcgaguccucgucauuuuuccgggccuguuuuuauacccuacuggacggagaacugauaagggcucgaauaggaccaagcaacauuugacguuaucugu
........................................................uggacggagCacugauaagg.....................................    6       1    Fm2
........................................................uggacggaUaacugauaagg.....................................    3       1    Fm2
........................................................uggacggUgaacugauaagg.....................................    12      1    Fm2
........................................................uggacNgagaacugauaagg.....................................    1       1    Fm2
........................................................uggacgUagaacugauaagg.....................................    1       1    Fm2
........................................................uggacggGgaacugauaagg.....................................    7       1    Fm2
........................................................uggacggagaacugauGagg.....................................    32      1    Fm2
........................................................uggaAggagaacugauaagg.....................................    3       1    Fm2
........................................................uggacUgagaacugauaagg.....................................    3       1    Fm2
........................................................Aggacggagaacugauaagg.....................................    16      1    Fm2
........................................................uggacgNagaacugauaagg.....................................    2       1    Fm2
........................................................uggacggagaacugauaagA.....................................    1695    1    Fm2
........................................................uggacggagaacuAauaagg.....................................    4       1    Fm2
........................................................uggCcggagaacugauaagg.....................................    5       1    Fm2
........................................................uggacggagaacugauaagU.....................................    534     1    Fm2
........................................................uAgacggagaacugauaagg.....................................    10      1    Fm2
........................................................uggacggagaacugauaaAg.....................................    124     1    Fm2
........................................................uggacAgagaacugauaagg.....................................    5       1    Fm2
........................................................uggacggagaacGgauaagg.....................................    1       1    Fm2
........................................................uggaGggagaacugauaagg.....................................    1       1    Fm2
........................................................ugAacggagaacugauaagg.....................................    6       1    Fm2
........................................................uggacggagaGcugauaagg.....................................    103     1    Fm2
........................................................uggUcggagaacugauaagg.....................................    4       1    Fm2
........................................................uggacggagaaUugauaagg.....................................    4       1    Fm2
........................................................uggacggagaacugGuaagg.....................................    5       1    Fm2
........................................................uggacggagaacugauaaCg.....................................    1       1    Fm2
........................................................uggacggagaaGugauaagg.....................................    1       1    Fm2
........................................................uggacggaCaacugauaagg.....................................    3       1    Fm2
........................................................uggacggagUacugauaagg.....................................    7       1    Fm2
........................................................uggacgAagaacugauaagg.....................................    3       1    Fm2
........................................................uggacggaAaacugauaagg.....................................    1       1    Fm2
........................................................uggacggagaacugauaaUg.....................................    4       1    Fm2
........................................................uggacggagaacugUuaagg.....................................    4       1    Fm2
........................................................uggacgCagaacugauaagg.....................................    2       1    Fm2
........................................................uggacggagaacugaGaagg.....................................    2       1    Fm2
........................................................ugCacggagaacugauaagg.....................................    3       1    Fm2
........................................................uggacggagaacugauaCgg.....................................    93      1    Fm2
........................................................uggacggagaacugauaagC.....................................    3       1    Fm2
........................................................uggacggCgaacugauaagg.....................................    4       1    Fm2
........................................................uggacggagaacugauUagg.....................................    28      1    Fm2
........................................................Cggacggagaacugauaagg.....................................    6       1    Fm2
........................................................uggaUggagaacugauaagg.....................................    6       1    Fm2
........................................................uggacggagaacuUauaagg.....................................    5       1    Fm2
........................................................uggacggagaCcugauaagg.....................................    68      1    Fm2
........................................................uggacggagaacCgauaagg.....................................    8       1    Fm2
........................................................uggacggagaacugauaGgg.....................................    112     1    Fm2
........................................................uggacggagaacugaCaagg.....................................    1       1    Fm2
........................................................uggacggagaacugauaagg.....................................    23066   0    Fm2
........................................................uggacggagGacugauaagg.....................................    23      1    Fm2
........................................................Nggacggagaacugauaagg.....................................    11      1    Fm2
........................................................uggacggagaacuCauaagg.....................................    2       1    Fm2
........................................................uggacggaAaacugauaaggg....................................    2       1    Fm2
........................................................uggacggagaacugauaagUg....................................    9       1    Fm2
........................................................uggacggagaacuUauaaggg....................................    2       1    Fm2
........................................................uggacAgagaacugauaaggg....................................    7       1    Fm2
........................................................uggacggagaacugauaaCgg....................................    3       1    Fm2
........................................................uggacCgagaacugauaaggg....................................    1       1    Fm2
........................................................uggacggagaacugaCaaggg....................................    6       1    Fm2
........................................................uggacggagaacCgauaaggg....................................    7       1    Fm2
........................................................uggacggagaacugauaaUgg....................................    13      1    Fm2
........................................................uggacggagaacugauUaggg....................................    56      1    Fm2
........................................................uggacggagaacugCuaaggg....................................    2       1    Fm2
........................................................uUgacggagaacugauaaggg....................................    10      1    Fm2
........................................................uggacggagaacugauaUggg....................................    112     1    Fm2
........................................................uggacggagGacugauaaggg....................................    27      1    Fm2
........................................................uggacggagaacugauGaggg....................................    52      1    Fm2
........................................................ugCacggagaacugauaaggg....................................    4       1    Fm2
........................................................uggacggagaacugaAaaggg....................................    2       1    Fm2
........................................................Cggacggagaacugauaaggg....................................    8       1    Fm2
........................................................uggacgCagaacugauaaggg....................................    3       1    Fm2
```

```
                    Star                              Mature
gaauuuguccgaaagcgaguccucgucauuuuuccgggccuguuuuuauacccuacuggacggagaacugauaagggcucgaauaggaccaagcaacauuugacguuaucugu
........................................................uAgacggagaacugauaaggg....................................   20     1   Fm2
........................................................uggacggagaacAgauaaggg....................................   3      1   Fm2
........................................................uggacggagaacugGuaaggg....................................   4      1   Fm2
........................................................uggCcggagaacugauaaggg....................................   4      1   Fm2
........................................................ugUacggagaacugauaaggg....................................   5      1   Fm2
........................................................uggacggCgaacugauaaggg....................................   2      1   Fm2
........................................................uggacggagaacugauCaggg....................................   75     1   Fm2
........................................................uggacggagaacugauaaAgg....................................   3      1   Fm2
........................................................uggacggagaacuAauaaggg....................................   3      1   Fm2
........................................................uggacggagaacugUuaaggg....................................   6      1   Fm2
........................................................uggacggagaGcugauaaggg....................................   127    1   Fm2
........................................................uggacggagaaAugauaaggg....................................   1      1   Fm2
........................................................uCgacggagaacugauaaggg....................................   3      1   Fm2
........................................................uggacggagUacugauaaggg....................................   16     1   Fm2
........................................................Gggacggagaacugauaaggg....................................   4      1   Fm2
........................................................uggacggagaacugauaCggg....................................   130    1   Fm2
........................................................Nggacggagaacugauaaggg....................................   16     1   Fm2
........................................................uggacggUgaacugauaaggg....................................   21     1   Fm2
........................................................uggUcggagaacugauaaggg....................................   4      1   Fm2
........................................................uggacggagaacuCauaaggg....................................   2      1   Fm2
........................................................uggacggagaaGugauaaggg....................................   1      1   Fm2
........................................................uggacggagaaUugauaaggg....................................   15     1   Fm2
........................................................uggacggagaCcugauaaggg....................................   67     1   Fm2
........................................................uggacgNagaacugauaaggg....................................   4      1   Fm2
........................................................uggacgAagaacugauaaggg....................................   2      1   Fm2
........................................................uggacggagaacugauaaggU....................................   824    1   Fm2
........................................................uggGcggagaacugauaaggg....................................   5      1   Fm2
........................................................uggaGggagaacugauaaggg....................................   5      1   Fm2
........................................................uggacggagaacugauaGggg....................................   193    1   Fm2
........................................................uggacggagCacugauaaggg....................................   8      1   Fm2
........................................................uggacgUagaacugauaaggg....................................   15     1   Fm2
........................................................uggacggagaacugauaagCg....................................   2      1   Fm2
........................................................uggacggagaacugauaaggg....................................   35348  0   Fm2
........................................................uggacggagaacugaGaaggg....................................   1      1   Fm2
........................................................uggacggagaacugauaaggC....................................   16     1   Fm2
........................................................uggaAggagaacugauaaggg....................................   2      1   Fm2
........................................................uggacggagaacugauaagAg....................................   8      1   Fm2
........................................................uggaUggagaacugauaaggg....................................   8      1   Fm2
........................................................uggacggagaacugauaaggA....................................   6724   1   Fm2
........................................................uggacUgagaacugauaaggg....................................   9      1   Fm2
........................................................Aggacggagaacugauaaggg....................................   22     1   Fm2
........................................................uggacNgagaacugauaaggg....................................   4      1   Fm2
........................................................ugAacggagaacugauaaggg....................................   8      1   Fm2
........................................................uggacggagaacugauaNggg....................................   1      1   Fm2
........................................................uggacggGgaacugauaaggg....................................   4      1   Fm2
........................................................uggacggaUaacugauaaggg....................................   5      1   Fm2
........................................................uggacUgagaacugauaagggc...................................   58     1   Fm2
........................................................uNgacggagaacugauaagggc...................................   3      1   Fm2
........................................................uggUcggagaacugauaagggc...................................   43     1   Fm2
........................................................ugCacggagaacugauaagggc...................................   28     1   Fm2
........................................................uggacggagaacugauaaUggc...................................   66     1   Fm2
........................................................uggacggagaacugCuaagggc...................................   7      1   Fm2
........................................................ugAacggagaacugauaagggc...................................   70     1   Fm2
........................................................uggCcggagaacugauaagggc...................................   44     1   Fm2
........................................................uggaGggagaacugauaagggc...................................   13     1   Fm2
........................................................uggacggagaacugauCagggc...................................   583    1   Fm2
........................................................uggacggagaacugauaagggG...................................   452    1   Fm2
........................................................ugUacggagaacugauaagggc...................................   54     1   Fm2
........................................................uggacggagaacuUauaagggc...................................   30     1   Fm2
........................................................uggacggagGacugauaagggc...................................   1032   1   Fm2
........................................................ugNacggagaacugauaagggc...................................   2      1   Fm2
........................................................uggacggagaacGgauaagggc...................................   7      1   Fm2
........................................................Nggacggagaacugauaagggc...................................   143    1   Fm2
........................................................uggacggagaaUugauaagggc...................................   70     1   Fm2
........................................................uggacggagaacugauaaggCc...................................   31     1   Fm2
........................................................uggacggaAaacugauaagggc...................................   15     1   Fm2
........................................................uggacggagaacugauUagggc...................................   345    1   Fm2
........................................................uggacggagaacugauaagCgc...................................   17     1   Fm2
........................................................uggacggagaacugauaaggAc...................................   83     1   Fm2
........................................................uggacgNagaacugauaagggc...................................   16     1   Fm2
```

Star Mature

gaauuuguccgaaagcgaguccucgucauuuuuccgggccuguuuuuauacccuacuggacggagaacugauaagggcucgaauaggaccaagcaacauuugacguuaucugu			
....uggacgAagaacugauaagggc...................................	32	1	Fm2
....uggacggagaacuCauaagggc...................................	16	1	Fm2
....uggacgCagaacugauaagggc...................................	27	1	Fm2
....uggacggagaacugaAaagggc...................................	7	1	Fm2
....uggacAgagaacugauaagggc...................................	38	1	Fm2
....uggacggagaacugauaagUgc...................................	78	1	Fm2
....uggacggagaGcugauaagggc...................................	478	1	Fm2
....Cggacggagaacugauaagggc...................................	37	1	Fm2
....uggacggGgaacugauaagggc...................................	83	1	Fm2
....uggacggagaaAugauaagggc...................................	5	1	Fm2
....uggacggagaacugauaUgggc...................................	904	1	Fm2
....uggacggaCaacugauaagggc...................................	11	1	Fm2
....uggacggagaCcugauaagggc...................................	221	1	Fm2
....uggacggagaacugaGaagggc...................................	9	1	Fm2
....uggaUggagaacugauaagggc...................................	84	1	Fm2
....Gggacggagaacugauaagggc...................................	7	1	Fm2
....uggacggagaacugUuaagggc...................................	40	1	Fm2
....uggacggagaaGugauaagggc...................................	6	1	Fm2
....uggacggagaacugauaagAgc...................................	38	1	Fm2
....uggacggCgaacugauaagggc...................................	50	1	Fm2
....uggacggagaacugauGagggc...................................	490	1	Fm2
....uAgacggagaacugauaagggc...................................	133	1	Fm2
....uggacggagCacugauaagggc...................................	56	1	Fm2
....uggacggagaacuAauaagggc...................................	64	1	Fm2
....uggacggagaacugauaaggUc...................................	78	1	Fm2
....uggacggagaacugauaagggc...................................	272144	0	Fm2
....uggacgUagaacugauaagggc...................................	60	1	Fm2
....uggacggagaacugauaaCggc...................................	8	1	Fm2
....Aggacggagaacugauaagggc...................................	62	1	Fm2
....uggacggagaacugauaGgggc...................................	6080	1	Fm2
....uggGcggagaacugauaagggc...................................	55	1	Fm2
....uUgacggagaacugauaagggc...................................	77	1	Fm2
....uggacggagaacugauaCgggc...................................	1038	1	Fm2
....uggacggagUacugauaagggc...................................	107	1	Fm2
....uggacggagaacugGuaagggc...................................	26	1	Fm2
....uggacggagaacugaCaagggc...................................	22	1	Fm2
....uggacggaUaacugauaagggc...................................	55	1	Fm2
....uggacCgagaacugauaagggc...................................	10	1	Fm2
....uggaAggagaacugauaagggc...................................	32	1	Fm2
....uggacggagaacugauaaAggc...................................	40	1	Fm2
....uggacggagaacugauaagggA...................................	13052	1	Fm2
....uggacggagaacugauaagggU...................................	2043	1	Fm2
....uggacNgagaacugauaagggc...................................	34	1	Fm2
....uggacggUgaacugauaagggc...................................	161	1	Fm2
....uggacggagaacAgauaagggc...................................	7	1	Fm2
....uggacggagaacCgauaagggc...................................	29	1	Fm2
....uCgacggagaacugauaagggc...................................	38	1	Fm2
....uggGcggagaacugauaagggcu..................................	1	1	Fm2
....uUgacggagaacugauaagggcu..................................	8	1	Fm2
....uggacggagaGcugauaagggcu..................................	18	1	Fm2
....uggaGggagaacugauaagggcu..................................	1	1	Fm2
....uggacggagaacugauaagggUu..................................	448	1	Fm2
....uggacAgagaacugauaagggcu..................................	3	1	Fm2
....uggacggagaacugUuaagggcu..................................	1	1	Fm2
....uggacggagaacuCauaagggcu..................................	1	1	Fm2
....Cggacggagaacugauaagggcu..................................	4	1	Fm2
....uggaAggagaacugauaagggcu..................................	1	1	Fm2
....uggacggagaCcugauaagggcu..................................	14	1	Fm2
....uggacggagaacugauUagggcu..................................	20	1	Fm2
....Nggacggagaacugauaagggcu..................................	4	1	Fm2
....uggacggagaacugauaaggCcu..................................	3	1	Fm2
....uggacggagaacugauaagUgcu..................................	4	1	Fm2
....uAgacggagaacugauaagggcu..................................	5	1	Fm2
....uggacggagaacugauaUgggcu..................................	46	1	Fm2
....uggacgAagaacugauaagggcu..................................	3	1	Fm2
....uggacggagaacugauaagggcu..................................	12261	0	Fm2
....uggacggagaacugauaagggcC..................................	179	1	Fm2
....uggacggagaacuUauaagggcu..................................	2	1	Fm2
....uggacggagaacugGuaagggcu..................................	2	1	Fm2
....uggacggagaaUugauaagggcu..................................	2	1	Fm2

```
                    Star                                   Mature
gaauuuguccgaaagcgaguccucgucauuuuuccgggccuguuuuuauacccuacuggacggagaacugauaagggcucgaauaggaccaagcaacauuugacguuaucugu
........................................................uggacggagaacugauaCgggcu..................................  44   1  Fm2
........................................................uNgacggagaacugauaagggcu..................................  1    1  Fm2
........................................................uggacggCgaacugauaagggcu..................................  2    1  Fm2
........................................................uggacggagaacugauaagggcG...................................  874  1  Fm2
........................................................uggacggagGacugauaagggcu..................................  37   1  Fm2
........................................................uggacggagaacugauaagCgcu..................................  1    1  Fm2
........................................................uggaUggagaacugauaagggcu..................................  2    1  Fm2
........................................................uggacggagaacAgauaagggcu..................................  1    1  Fm2
........................................................uggacggagaacugaAaagggcu..................................  2    1  Fm2
........................................................Aggacggagaacugauaagggcu..................................  2    1  Fm2
........................................................uggacggUgaacugauaagggcu..................................  8    1  Fm2
........................................................uggacggagaacugauaaUggcu..................................  4    1  Fm2
........................................................uCgacggagaacugauaagggcu..................................  1    1  Fm2
........................................................ugUacggagaacugauaagggcu..................................  2    1  Fm2
........................................................uggacggagaacugauaagAgcu..................................  2    1  Fm2
........................................................uggacggagaacugauaaggAcu..................................  4    1  Fm2
........................................................uggCcggagaacugauaagggcu..................................  1    1  Fm2
........................................................uggacgUagaacugauaagggcu..................................  3    1  Fm2
........................................................uggacggagaacugauCagggcu..................................  29   1  Fm2
........................................................uggacgNagaacugauaagggcu..................................  1    1  Fm2
........................................................uggacggagUacugauaagggcu..................................  3    1  Fm2
........................................................ugCacggagaacugauaagggcu..................................  1    1  Fm2
........................................................uggacggagaacugauaaAggcu..................................  11   1  Fm2
........................................................uggUcggagaacugauaagggcu..................................  3    1  Fm2
........................................................uggacggagaacugauGagggcu..................................  32   1  Fm2
........................................................uggacNgagaacugauaagggcu..................................  2    1  Fm2
........................................................uggacggagCacugauaagggcu..................................  3    1  Fm2
........................................................uggacggagaacugauaagggAu..................................  634  1  Fm2
........................................................uggacggaUaacugauaagggcu..................................  2    1  Fm2
........................................................uggacggGgaacugauaagggcu..................................  1    1  Fm2
........................................................uggacggagaacugauaaggUcu..................................  5    1  Fm2
........................................................uggacUgagaacugauaagggcu..................................  4    1  Fm2
........................................................uggacggagaacuAauaagggcu..................................  2    1  Fm2
........................................................uggacggagaacugauaGgggcu..................................  292  1  Fm2
........................................................uggacggagaacugauaagggcuA.................................  113  1  Fm2
........................................................uggacggagaacugauaCgggcuc.................................  1    1  Fm2
........................................................uggacggagaacugauaagggcuG.................................  31   1  Fm2
........................................................uggacggagaacugauaagggcuc.................................  108  0  Fm2
........................................................uggacggagaacugauaGgggcuc.................................  2    1  Fm2
.........................................................ggacggagaacugauaaA......................................  2    1  Fm2
.........................................................ggacggagaacugauaaU......................................  5    1  Fm2
.........................................................ggacggagaacugauaag......................................  3    0  Fm2
.........................................................ggacggagGacugauaagg.....................................  1    1  Fm2
.........................................................ggacggagaacugauaagg.....................................  30   0  Fm2
.........................................................ggacggagaacugauaagU.....................................  1    1  Fm2
.........................................................ggacggagaacugauaagA.....................................  2    1  Fm2
.........................................................ggacggagaacGgauaagg.....................................  1    1  Fm2
.........................................................ggacggagaacugauaGgg.....................................  1    1  Fm2
.........................................................Ugacggagaacugauaagg.....................................  2    1  Fm2
.........................................................ggacggagaacugauaaggA....................................  8    1  Fm2
.........................................................ggacggagaacugauaagAg....................................  1    1  Fm2
.........................................................Ugacggagaacugauaaggg....................................  5    1  Fm2
.........................................................ggacggagaacugauaaggg....................................  39   0  Fm2
.........................................................ggacggagaacugauaagUg....................................  1    1  Fm2
.........................................................ggacggagaacugauaagCgc...................................  1    1  Fm2
.........................................................ggacggagUacugauaagggc...................................  1    1  Fm2
.........................................................ggacggagaacugauaGgggc...................................  6    1  Fm2
.........................................................ggacggagaacugauaagggc...................................  191  0  Fm2
.........................................................ggacggagaacugauaaggAc...................................  3    1  Fm2
.........................................................ggacggagaacugauaaggCc...................................  1    1  Fm2
.........................................................Ugacggagaacugauaagggc...................................  13   1  Fm2
.........................................................ggacggagaaAugauaagggc...................................  1    1  Fm2
.........................................................ggacggNgaacugauaagggc...................................  1    1  Fm2
.........................................................ggacgUagaacugauaagggc...................................  1    1  Fm2
.........................................................ggacggagaacuAauaagggc...................................  2    1  Fm2
.........................................................ggacggagaaUugauaagggc...................................  2    1  Fm2
.........................................................ggacggagCacugauaagggc...................................  3    1  Fm2
.........................................................Agacggagaacugauaagggc...................................  1    1  Fm2
.........................................................ggacggagaacugauaagggA...................................  22   1  Fm2
.........................................................ggacggagaacugauaagggU...................................  1    1  Fm2
```

Star Mature

gaauuuguccgaaagcgaguccucgucauuuuuccgggccuguuuuuauacccuacuggacggagaacugauaagggcucgaauaggaccaagcaacauuugacguuaucugu			
....ggacggUgaacugauaagggc...................................	1	1	Fm2
....ggacggaAaacugauaagggc...................................	1	1	Fm2
....ggacggagaacuUauaagggc...................................	1	1	Fm2
....ggacggagaacugauaagggcu..................................	8	0	Fm2
....ggacggCgaacugauaagggcu..................................	1	1	Fm2
....ggacggagaacugauaagggcG..................................	1	1	Fm2
....ggacggagaacuUauaagggcuc.................................	1	1	Fm2
....ggacggagaacugauaagggcuc.................................	2	0	Fm2
....gacggagaacugauaagg.....................................	1	0	Fm2
....cggagaacugauaagggc...................................	5	0	Fm2
....Uggagaacugauaagggc...................................	1	1	Fm2
...................uccucgucauuuuuccgggccugu..	3	0	Fm1
....................ccucgucauuuuuccggg...	4	0	Fm1
....................ccucgucauuuuuccgggcc...	2	0	Fm1
....................ccucgucauuuuuccggCccu..	1	1	Fm1
....................ccucgucauuuuuccgggccu..	5	0	Fm1
....................ccucgucauuuuuccgggccGg...	1	1	Fm1
....................ccucgucauuuuuccgggccug...	9	0	Fm1
....................ccucgucauuuuuccgggcUugu..	1	1	Fm1
....................Ucucgucauuuuuccgggccugu..	7	1	Fm1
....................ccucgucauuuuuccgggccugC..	1	1	Fm1
....................ccucgucauuCuuccgggccugu..	1	1	Fm1
....................Ncucgucauuuuuccgggccugu..	1	1	Fm1
....................ccGcgucauuuuuccgggccugu..	1	1	Fm1
....................ccucgucauuuuuUcgggccugu..	1	1	Fm1
....................ccucgucauuuuuccgggccugA..	6	1	Fm1
....................ccucgucauuuuuccggUccugu..	6	1	Fm1
....................ccucgucauuuuuccUggccugu..	1	1	Fm1
....................ccucgucauuuuuccgggccugG..	20	1	Fm1
....................ccucgucauuuuuccggCccugu..	2	1	Fm1
....................ccucgucauuuuuccgggccCgu..	1	1	Fm1
....................ccucgucauuuuuccggAccugu..	1	1	Fm1
....................ccucgucauuuuuccgggccugu..	730	0	Fm1
....................ccucgucauuuuCccgggccugu..	2	1	Fm1
....................ccucgucauuGuuccgggccugu..	1	1	Fm1
....................ccucgucaAuuuuccgggccugu..	1	1	Fm1
....................ccucgucauuuuuccgAgccugu..	1	1	Fm1
....................ccucgucauuAuuccgggccugu..	1	1	Fm1
....................ccucgucauuuuuccgCgccugu..	2	1	Fm1
....................ccucgucGuuuuuccgggccugu..	1	1	Fm1
....................ccAcgucauuuuuccgggccugu..	1	1	Fm1
....................ccucgucauuuuuccgUgccugu..	3	1	Fm1
....................ccuUgucauuuuuccgggccugu..	2	1	Fm1
....................ccucgucauuuuuccgggccuguA...	3	1	Fm1
....................ccucgucauuuuuccgggccuguu...	75	0	Fm1
....................Ucucgucauuuuuccgggccuguu...	2	1	Fm1
....................ccucgucauuuuuccgggccuguuu..	16	0	Fm1
....................ccucgucauuuuuccgggccuguuuu...	2	0	Fm1
.....................cucgucauuuuuccgggccugu..	17	0	Fm1
.....................cucgucauuuuuccgggccuguu...	2	0	Fm1
.....................cucgucauuuuuccgggccuguuu..	4	0	Fm1
.......................cgucauuuuuccgggccugu..	2	0	Fm1
....uuuauacccuacuggacggagaacugau...	1	0	Fm1
....acuggacggagaacugauaaU......................................	4	1	Fm1
....acuggacggagaacugauaagg.....................................	2	0	Fm1
....acuggacggagaacugauaaggA....................................	2	1	Fm1
....acuggacggagaacugauaagggc...................................	3	0	Fm1
....cuggacggagaacugaua..	2	0	Fm1
....cuggacggagaacugauCa.......................................	1	1	Fm1
....cuggacggagaacugaAaa.......................................	5	1	Fm1
....cuggacggagGacugauaa.......................................	1	1	Fm1
....cuggacggagaacugauaa.......................................	6	0	Fm1
....cuggacggagaacugauaag......................................	36	0	Fm1
....cuggacggagaacugauaaU......................................	3	1	Fm1
....cuggacggagaacugauaaA......................................	14	1	Fm1
....cAggacggagaacugauaag......................................	1	1	Fm1
....cuggaNggagaacugauaagg.....................................	1	1	Fm1
....cuggacggagaacugauaagU.....................................	1	1	Fm1
....cuggacggagaacugauCagg.....................................	1	1	Fm1

```
                    Star                         Mature
gaauuuguccgaaagcgaguccucgucauuuuuccgggccuguuuuuauacccuacuggacggagaacugauaagggcucgaauaggaccaagcaacauuugacguuaucugu
.......................................................cuggacggaUaacugauaagg.....................................    1      1    Fm1
.......................................................cuggacggagaacugauaagA.....................................    18     1    Fm1
.......................................................cuggacggagaacAgauaagg.....................................    1      1    Fm1
.......................................................cuggacggagaacugauaagg.....................................    165    0    Fm1
.......................................................Nuggacggagaacugauaagg.....................................    1      1    Fm1
.......................................................cuggacUgagaacugauaaggg....................................    1      1    Fm1
.......................................................cuggacggagaacugauaaggg....................................    169    0    Fm1
.......................................................cuggacggagaacugauGaggg....................................    2      1    Fm1
.......................................................cuggacggagaacugauaaggU....................................    32     1    Fm1
.......................................................cuggacggagCacugauaaggg....................................    1      1    Fm1
.......................................................cuggacggagaacugauaaggC....................................    5      1    Fm1
.......................................................cuggacggagaacugauaaggA....................................    15     1    Fm1
.......................................................cuggacggagaacugaGaaggg....................................    1      1    Fm1
.......................................................cuggacggagUacugauaaggg....................................    1      1    Fm1
.......................................................cuggacggagaacugaAaagggc...................................    1      1    Fm1
.......................................................cuggacggagaacugauaagggU...................................    4      1    Fm1
.......................................................cuggacggagaacugauGagggc...................................    1      1    Fm1
.......................................................cuggacggagaCcugauaagggc...................................    1      1    Fm1
.......................................................cuggacggagUacugauaagggc...................................    2      1    Fm1
.......................................................cuggacggGgaacugauaagggc...................................    2      1    Fm1
.......................................................cuggacggUgaacugauaagggc...................................    1      1    Fm1
.......................................................cuggacggagaaUugauaagggc...................................    1      1    Fm1
.......................................................cuggacggagaacugauUagggc...................................    1      1    Fm1
.......................................................cuggacggagaacugauCagggc...................................    1      1    Fm1
.......................................................Nuggacggagaacugauaagggc...................................    1      1    Fm1
.......................................................cuggacggagaacugaCaagggc...................................    4      1    Fm1
.......................................................cuggacggagaacugauaagggc...................................    422    0    Fm1
.......................................................cuggacggagGacugauaagggc...................................    4      1    Fm1
.......................................................cuggacAgagaacugauaagggc...................................    1      1    Fm1
.......................................................cuggacUgagaacugauaagggc...................................    1      1    Fm1
.......................................................Uuggacggagaacugauaagggc...................................    1      1    Fm1
.......................................................cuggacggagaacugauaGgggc...................................    9      1    Fm1
.......................................................cuggacggagaacugauaagggA...................................    37     1    Fm1
.......................................................cGggacggagaacugauaagggc...................................    1      1    Fm1
.......................................................cuggacggagaacugauaagggcu..................................    14     0    Fm1
.......................................................cuggacggagaacugauaagggAu..................................    5      1    Fm1
.......................................................cuggacggagaacuAauaagggcu..................................    1      1    Fm1
.......................................................cuggacggagaacugauaagggcG..................................    1      1    Fm1
........................................................uggacggagaacugaGaa.......................................    6      1    Fm1
........................................................uggacggagaacugauaC.......................................    15     1    Fm1
........................................................uAgacggagaacugauaa.......................................    2      1    Fm1
........................................................uggacggagaCcugauaa.......................................    1      1    Fm1
........................................................uggacggagUacugauaa.......................................    1      1    Fm1
........................................................uggacggagaacugauGa.......................................    1      1    Fm1
........................................................uggacggagaacugauaa.......................................    2454   0    Fm1
........................................................uggacggagaacugauCa.......................................    3      1    Fm1
........................................................uggacggagaacugUuaa.......................................    1      1    Fm1
........................................................ugAacggagaacugauaa.......................................    1      1    Fm1
........................................................uggacggagaGcugauaa.......................................    10     1    Fm1
........................................................uggacggagaacugaAaa.......................................    215    1    Fm1
........................................................uggGcggagaacugauaa.......................................    1      1    Fm1
........................................................uggacggUgaacugauaa.......................................    2      1    Fm1
........................................................uggacggagaacugauaU.......................................    88     1    Fm1
........................................................Nggacggagaacugauaa.......................................    1      1    Fm1
........................................................uggacggagaacugauaG.......................................    14     1    Fm1
........................................................uggacggagaacugCuaa.......................................    1      1    Fm1
........................................................uggacggagaacugauUa.......................................    9      1    Fm1
........................................................uggaUggagaacugauaag......................................    4      1    Fm1
........................................................uggacggagaacuAauaag......................................    2      1    Fm1
........................................................Nggacggagaacugauaag......................................    10     1    Fm1
........................................................uggacggUgaacugauaag......................................    4      1    Fm1
........................................................uggacggaUaacugauaag......................................    4      1    Fm1
........................................................uggaAggagaacugauaag......................................    1      1    Fm1
........................................................uggacggagaacugauaaU......................................    4300   1    Fm1
........................................................uggGcggagaacugauaag......................................    8      1    Fm1
........................................................uggacggagGacugauaag......................................    9      1    Fm1
........................................................ugCacggagaacugauaag......................................    2      1    Fm1
........................................................uggacggagaacugauaGg......................................    72     1    Fm1
........................................................uggacggagCacugauaag......................................    2      1    Fm1
........................................................uggUcggagaacugauaag......................................    5      1    Fm1
```

```
                          Star                                      Mature
gaauuuguccgaaagcgaguccucgucauuuuuccgggccuguuuuuauacccuacuggacggagaacugauaagggcucgaauaggaccaagcaacauuugacguuaucugu
........................................................uggacggagaacuUauaag......................................    3      1     Fm1
........................................................uggacggagUacugauaag......................................    3      1     Fm1
........................................................uggacggagaacGgauaag......................................    1      1     Fm1
........................................................uggacggagaacugauaag......................................    17744  0     Fm1
........................................................uAgacggagaacugauaag......................................    10     1     Fm1
........................................................uggacggagaacugCuaag......................................    1      1     Fm1
........................................................uggacggagaacugauaCg......................................    70     1     Fm1
........................................................uggacggagaacugauUag......................................    24     1     Fm1
........................................................uggacNgagaacugauaag......................................    4      1     Fm1
........................................................uggacggGgaacugauaag......................................    4      1     Fm1
........................................................uggacgAagaacugauaag......................................    3      1     Fm1
........................................................uggacgUagaacugauaag......................................    5      1     Fm1
........................................................uggacggagaacugUuaag......................................    6      1     Fm1
........................................................uggacUgagaacugauaag......................................    5      1     Fm1
........................................................uggacggagaaUugauaag......................................    8      1     Fm1
........................................................uggacggaAaacugauaag......................................    3      1     Fm1
........................................................uggCcggagaacugauaag......................................    1      1     Fm1
........................................................Cggacggagaacugauaag......................................    2      1     Fm1
........................................................ugUacggagaacugauaag......................................    1      1     Fm1
........................................................uggacggagaacAgauaag......................................    1      1     Fm1
........................................................uggacggagaacugauaaC......................................    30     1     Fm1
........................................................ugAacggagaacugauaag......................................    5      1     Fm1
........................................................Aggacggagaacugauaag......................................    3      1     Fm1
........................................................uggacggagaacugauaUg......................................    63     1     Fm1
........................................................uggacggagaaAugauaag......................................    1      1     Fm1
........................................................uNgacggagaacugauaag......................................    1      1     Fm1
........................................................uggacggagaacCgauaag......................................    4      1     Fm1
........................................................uggacgCagaacugauaag......................................    1      1     Fm1
........................................................uggacggagaacugauGag......................................    21     1     Fm1
........................................................uggacggagaGcugauaag......................................    31     1     Fm1
........................................................uggacggagaacugaCaag......................................    2      1     Fm1
........................................................uggacggagaacugauaaA......................................    6927   1     Fm1
........................................................uggaGggagaacugauaag......................................    1      1     Fm1
........................................................uggacAgagaacugauaag......................................    5      1     Fm1
........................................................uggacggagaacugaAaag......................................    19     1     Fm1
........................................................uggacggagaCcugauaag......................................    24     1     Fm1
........................................................uggacggagaacugauCag......................................    39     1     Fm1
........................................................uUgacggagaacugauaag......................................    1      1     Fm1
........................................................uggacggagaacuCauaag......................................    2      1     Fm1
........................................................uggacCgagaacugauaag......................................    1      1     Fm1
........................................................uggacggagaacugUuaagg.....................................    9      1     Fm1
........................................................uggacggagaacugauaagg.....................................    46362  0     Fm1
........................................................uggacggagaacugauUagg.....................................    70     1     Fm1
........................................................ugUacggagaacugauaagg.....................................    5      1     Fm1
........................................................Cggacggagaacugauaagg.....................................    9      1     Fm1
........................................................uggGcggagaacugauaagg.....................................    16     1     Fm1
........................................................uggCcggagaacugauaagg.....................................    9      1     Fm1
........................................................Aggacggagaacugauaagg.....................................    17     1     Fm1
........................................................uggacUgagaacugauaagg.....................................    14     1     Fm1
........................................................uggacgAagaacugauaagg.....................................    4      1     Fm1
........................................................uggacggagGacugauaagg.....................................    51     1     Fm1
........................................................uggacggagaacugauaagU.....................................    1110   1     Fm1
........................................................uUgacggagaacugauaagg.....................................    8      1     Fm1
........................................................uggacggagaacugauaagC.....................................    31     1     Fm1
........................................................uggUcggagaacugauaagg.....................................    8      1     Fm1
........................................................uggacggagaacuCauaagg.....................................    4      1     Fm1
........................................................uggacgCagaacugauaagg.....................................    2      1     Fm1
........................................................uggacggagaacugauaaAg.....................................    266    1     Fm1
........................................................uggacggagaacugauaaUg.....................................    3      1     Fm1
........................................................uggacggagaacugaCaagg.....................................    2      1     Fm1
........................................................uggacggagaacugCuaagg.....................................    2      1     Fm1
........................................................ugAacggagaacugauaagg.....................................    9      1     Fm1
........................................................uggacggagaacugaAaagg.....................................    2      1     Fm1
........................................................uggacggaCaacugauaagg.....................................    1      1     Fm1
........................................................uggacggagaaGugauaagg.....................................    1      1     Fm1
........................................................uggacggaAaacugauaagg.....................................    2      1     Fm1
........................................................uggacggagaacugauaaCg.....................................    4      1     Fm1
........................................................uggacggagaacuUauaagg.....................................    1      1     Fm1
........................................................uggacggagaacugauaCgg.....................................    177    1     Fm1
........................................................uggacggagaacuAauaagg.....................................    9      1     Fm1
```

Star   Mature

```
gaauuuguccgaaagcgaguccucgucauuuuuccgggccuguuuuuauacccuacuggacggagaacugauaagggcucgaauaggaccaagcaacauuugacguuaucugu
........................................................uNgacggagaacugauaagg.....................................    2      1    Fm1
........................................................uggacggagaacugauaagA.....................................    4462   1    Fm1
........................................................uggacAgagaacugauaagg.....................................    5      1    Fm1
........................................................uggacgNagaacugauaagg.....................................    2      1    Fm1
........................................................uggacNgagaacugauaagg.....................................    2      1    Fm1
........................................................uggacggagaGcugauaagg.....................................    149    1    Fm1
........................................................uggacggGgaacugauaagg.....................................    8      1    Fm1
........................................................uggacggUgaacugauaagg.....................................    18     1    Fm1
........................................................uggacggagCacugauaagg.....................................    6      1    Fm1
........................................................uggacggagaacugauCagg.....................................    122    1    Fm1
........................................................uggacggagUacugauaagg.....................................    17     1    Fm1
........................................................uAgacggagaacugauaagg.....................................    27     1    Fm1
........................................................uCgacggagaacugauaagg.....................................    4      1    Fm1
........................................................uggacggagaacugauGagg.....................................    63     1    Fm1
........................................................uggacgUagaacugauaagg.....................................    8      1    Fm1
........................................................uggacggagaacAgauaagg.....................................    2      1    Fm1
........................................................uggacggagaacugGuaagg.....................................    7      1    Fm1
........................................................uggacggCgaacugauaagg.....................................    4      1    Fm1
........................................................uggacggagaacugauaGgg.....................................    199    1    Fm1
........................................................ugCacggagaacugauaagg.....................................    1      1    Fm1
........................................................uggacggagaCcugauaagg.....................................    71     1    Fm1
........................................................uggacggaUaacugauaagg.....................................    8      1    Fm1
........................................................uggacggagaacugauaUgg.....................................    165    1    Fm1
........................................................uggacggagaaUugauaagg.....................................    8      1    Fm1
........................................................uggaAggagaacugauaagg.....................................    1      1    Fm1
........................................................Nggacggagaacugauaagg.....................................    17     1    Fm1
........................................................uggacCgagaacugauaagg.....................................    2      1    Fm1
........................................................uggacggagaacCgauaagg.....................................    3      1    Fm1
........................................................uggaUggagaacugauaagg.....................................    9      1    Fm1
........................................................uggacggagaacugauaaggA....................................    11343  1    Fm1
........................................................uggacggaUaacugauaaggg....................................    14     1    Fm1
........................................................uggacNgagaacugauaaggg....................................    11     1    Fm1
........................................................uggacgUagaacugauaaggg....................................    16     1    Fm1
........................................................uggacggagaacGgauaaggg....................................    1      1    Fm1
........................................................uggGcggagaacugauaaggg....................................    14     1    Fm1
........................................................uggacUgagaacugauaaggg....................................    15     1    Fm1
........................................................uggacgCagaacugauaaggg....................................    3      1    Fm1
........................................................uggUcggagaacugauaaggg....................................    6      1    Fm1
........................................................uggCcggagaacugauaaggg....................................    10     1    Fm1
........................................................uggacggGgaacugauaaggg....................................    18     1    Fm1
........................................................uggacggagaacugCuaaggg....................................    4      1    Fm1
........................................................uggacggagCacugauaaggg....................................    8      1    Fm1
........................................................uggacggagaacAgauaaggg....................................    4      1    Fm1
........................................................uggacCgagaacugauaaggg....................................    4      1    Fm1
........................................................uggacggagaacugauCaggg....................................    154    1    Fm1
........................................................uggacggagaacugauaGggg....................................    524    1    Fm1
........................................................uggacgNagaacugauaaggg....................................    5      1    Fm1
........................................................uggacggagaacuAauaaggg....................................    13     1    Fm1
........................................................uggacggagaacugaGaaggg....................................    2      1    Fm1
........................................................uggacggagaacugauaagAg....................................    22     1    Fm1
........................................................uggacggagaacugUuaaggg....................................    11     1    Fm1
........................................................uggacggagaacugauUaggg....................................    93     1    Fm1
........................................................uggacggagaacuUauaaggg....................................    4      1    Fm1
........................................................uggacggagGacugauaaggg....................................    59     1    Fm1
........................................................uggacggagaacugauaaAgg....................................    6      1    Fm1
........................................................uggacggagaacugauaagCg....................................    5      1    Fm1
........................................................uggacggaAaacugauaaggg....................................    2      1    Fm1
........................................................uggacggagaCcugauaaggg....................................    72     1    Fm1
........................................................Nggacggagaacugauaaggg....................................    26     1    Fm1
........................................................uggacggCgaacugauaaggg....................................    5      1    Fm1
........................................................uCgacggagaacugauaaggg....................................    5      1    Fm1
........................................................ugCacggagaacugauaaggg....................................    1      1    Fm1
........................................................Gggacggagaacugauaaggg....................................    1      1    Fm1
........................................................uggacggagaGcugauaaggg....................................    136    1    Fm1
........................................................uggacggagaacugauaaggU....................................    1894   1    Fm1
........................................................uggacggagaacugaCaaggg....................................    2      1    Fm1
........................................................uggacggagaacugauaagUg....................................    14     1    Fm1
........................................................uggacggagaacugGuaaggg....................................    13     1    Fm1
........................................................uggacggagaacugauaaggg....................................    58344  0    Fm1
........................................................uggaGggagaacugauaaggg....................................    3      1    Fm1
```

Star Mature

```
gaauuuguccgaaagcgaguccucgucauuuuuccgggccuguuuuuauacccuacuggacggagaacugauaagggcucgaauaggaccaagcaacauuugacguuaucugu
........................................................uUgacggagaacugauaaggg....................................   14      1   Fm1
........................................................uggacggagaacugauGaggg....................................   97      1   Fm1
........................................................uAgacggagaacugauaaggg....................................   30      1   Fm1
........................................................uggacgAagaacugauaaggg....................................   8       1   Fm1
........................................................Aggacggagaacugauaaggg....................................   24      1   Fm1
........................................................uggacggagaacugaAaaggg....................................   2       1   Fm1
........................................................uggacggagaacugauaCggg....................................   225     1   Fm1
........................................................Cggacggagaacugauaaggg....................................   9       1   Fm1
........................................................uggacAgagaacugauaaggg....................................   8       1   Fm1
........................................................ugUacggagaacugauaaggg....................................   3       1   Fm1
........................................................uggacggagaacugauaUggg....................................   223     1   Fm1
........................................................uggacggaCaacugauaaggg....................................   3       1   Fm1
........................................................uggacggagaacuCauaaggg....................................   5       1   Fm1
........................................................uggaAggagaacugauaaggg....................................   1       1   Fm1
........................................................uggaUggagaacugauaaggg....................................   13      1   Fm1
........................................................uggacggagaacugauaaggC....................................   348     1   Fm1
........................................................ugNacggagaacugauaaggg....................................   1       1   Fm1
........................................................uggacggagaaUugauaaggg....................................   20      1   Fm1
........................................................ugAacggagaacugauaaggg....................................   12      1   Fm1
........................................................uggacggagaacCgauaaggg....................................   12      1   Fm1
........................................................uggacggagUacugauaaggg....................................   19      1   Fm1
........................................................uggacggUgaacugauaaggg....................................   20      1   Fm1
........................................................uggacggagaacugauaaUgg....................................   8       1   Fm1
........................................................uggacggGgaacugauaagggc...................................   194     1   Fm1
........................................................Aggacggagaacugauaagggc...................................   153     1   Fm1
........................................................Nggacggagaacugauaagggc...................................   440     1   Fm1
........................................................uggacggagaacugauaagUgc...................................   175     1   Fm1
........................................................uggacNgagaacugauaagggc...................................   135     1   Fm1
........................................................uggacggagaCcugauaagggc...................................   672     1   Fm1
........................................................uggacggagaaAugauaagggc...................................   22      1   Fm1
........................................................ugNacggagaacugauaagggc...................................   4       1   Fm1
........................................................uggacggagaacugauaCgggc...................................   3205    1   Fm1
........................................................uggacggagaacugaCaagggc...................................   80      1   Fm1
........................................................uggacggagaacGgauaagggc...................................   33      1   Fm1
........................................................uggacggaAaacugauaagggc...................................   49      1   Fm1
........................................................uggacggagaacugauaagAgc...................................   127     1   Fm1
........................................................uggacggagaacugaGaagggc...................................   21      1   Fm1
........................................................uggacggagaacCgauaagggc...................................   121     1   Fm1
........................................................uggaGggagaacugauaagggc...................................   38      1   Fm1
........................................................uggUcggagaacugauaagggc...................................   135     1   Fm1
........................................................Gggacggagaacugauaagggc...................................   22      1   Fm1
........................................................uggacggagaGcugauaagggc...................................   1253    1   Fm1
........................................................uggacggagaacugauaaCggc...................................   16      1   Fm1
........................................................uggacggagaacugauaaAggc...................................   61      1   Fm1
........................................................uggacggagaacugauaagggc...................................   846342  0   Fm1
........................................................uggacggagaacugauGagggc...................................   1216    1   Fm1
........................................................uggacggagGacugauaagggc...................................   1684    1   Fm1
........................................................uggacggagaacugauUagggc...................................   1143    1   Fm1
........................................................uggacggagaaUugauaagggc...................................   284     1   Fm1
........................................................uCgacggagaacugauaagggc...................................   73      1   Fm1
........................................................uggacggaCaacugauaagggc...................................   44      1   Fm1
........................................................uggacggagaacAgauaagggc...................................   31      1   Fm1
........................................................uggacggagaacuAauaagggc...................................   170     1   Fm1
........................................................uggacggagaacugauaagCgc...................................   50      1   Fm1
........................................................uggacggagaacugauaagggA...................................   21574   1   Fm1
........................................................uggacCgagaacugauaagggc...................................   34      1   Fm1
........................................................uggacggagaaGugauaagggc...................................   18      1   Fm1
........................................................uggacggagaacugauaagggU...................................   7010    1   Fm1
........................................................uggacgNagaacugauaagggc...................................   46      1   Fm1
........................................................uggacggagCacugauaagggc...................................   169     1   Fm1
........................................................uggaUggagaacugauaagggc...................................   194     1   Fm1
........................................................uggacggagaacugauaUgggc...................................   2648    1   Fm1
........................................................uNgacggagaacugauaagggc...................................   7       1   Fm1
........................................................uggacggagaacugGuaagggc...................................   99      1   Fm1
........................................................uggacggagaacugauaNgggc...................................   2       1   Fm1
........................................................Cggacggagaacugauaagggc...................................   102     1   Fm1
........................................................uggacAgagaacugauaagggc...................................   137     1   Fm1
........................................................uAgacggagaacugauaagggc...................................   390     1   Fm1
........................................................uggacggagaacugauaaUggc...................................   128     1   Fm1
........................................................uggacggagaacugauaaggCc...................................   116     1   Fm1
```

Star **Mature**

gaauuuguccgaaagcgaguccucgucauuuuuccgggccuguuuuuauacccuacuggacggagaacugauaagggcucgaauaggaccaagcaacauuugacguuaucugu

....uggacggagaacugauaaggAc...................................	313	1	Fm1
....uggGcggagaacugauaagggc...................................	170	1	Fm1
....uggacgAagaacugauaagggc...................................	98	1	Fm1
....uggacggagaacugauaaggUc...................................	223	1	Fm1
....ugCacggagaacugauaagggc...................................	65	1	Fm1
....uggacggagaacugUuaagggc...................................	110	1	Fm1
....uggacggaUaacugauaagggc...................................	154	1	Fm1
....uUgacggagaacugauaagggc...................................	193	1	Fm1
....uggacggagaacugauCagggc...................................	1755	1	Fm1
....uggacggUgaacugauaagggc...................................	431	1	Fm1
....uggaAggagaacugauaagggc...................................	66	1	Fm1
....uggacggagaacugCuaagggc...................................	25	1	Fm1
....uggacgUagaacugauaagggc...................................	178	1	Fm1
....uggCcggagaacugauaagggc...................................	174	1	Fm1
....uggacggagaacugaAaagggc...................................	18	1	Fm1
....uggacggCgaacugauaagggc...................................	86	1	Fm1
....uggacggagaacugauaagggG...................................	874	1	Fm1
....uggacggagaacuUauaagggc...................................	78	1	Fm1
....ugAacggagaacugauaagggc...................................	183	1	Fm1
....uggacgCagaacugauaagggc...................................	47	1	Fm1
....uggacUgagaacugauaagggc...................................	145	1	Fm1
....ugUacggagaacugauaagggc...................................	131	1	Fm1
....uggacggagaacugauaGgggc...................................	8811	1	Fm1
....uggacggagaacuCauaagggc...................................	49	1	Fm1
....uggacggagUacugauaagggc...................................	282	1	Fm1
....uggacggagaacuCauaagggcu..................................	2	1	Fm1
....uggacggagaGcugauaagggcu..................................	58	1	Fm1
....uggacggagaacugauUagggcu..................................	56	1	Fm1
....uggacggagaacugauaCgggcu..................................	143	1	Fm1
....uggUcggagaacugauaagggcu..................................	9	1	Fm1
....Aggacggagaacugauaagggcu..................................	4	1	Fm1
....uggacggagaacugaCaagggcu..................................	5	1	Fm1
....uggacggagaacugGuaagggcu..................................	3	1	Fm1
....uggacggagaacAgauaagggcu..................................	1	1	Fm1
....uggacggagaacugauaaNggcu..................................	1	1	Fm1
....uggacggagaaGugauaagggcu..................................	1	1	Fm1
....uggacggagaCcugauaagggcu..................................	32	1	Fm1
....ugCacggagaacugauaagggcu..................................	3	1	Fm1
....uAgacggagaacugauaagggcu..................................	21	1	Fm1
....ugUacggagaacugauaagggcu..................................	9	1	Fm1
....uggGcggagaacugauaagggcu..................................	8	1	Fm1
....uggacggagUacugauaagggcu..................................	12	1	Fm1
....uggacggagaaUugauaagggcu..................................	12	1	Fm1
....uggacggagaacugauaagggGu..................................	13	1	Fm1
....uggacggagaacuAauaagggcu..................................	9	1	Fm1
....uggacggagaacugauaUgggcu..................................	138	1	Fm1
....uggCcggagaacugauaagggcu..................................	14	1	Fm1
....uggacggagaacugauaagAgcu..................................	7	1	Fm1
....uggacggagaacugauaagggAu..................................	1469	1	Fm1
....uggacggagCacugauaagggcu..................................	14	1	Fm1
....uggacUgagaacugauaagggcu..................................	5	1	Fm1
....uggaGggagaacugauaagggcu..................................	3	1	Fm1
....uggacggagaaAugauaagggcu..................................	2	1	Fm1
....uggacggagaacugUuaagggcu..................................	7	1	Fm1
....uggacggagaacugauaagggcC..................................	616	1	Fm1
....Nggacggagaacugauaagggcu..................................	14	1	Fm1
....uggacggagaacugCuaagggcu..................................	1	1	Fm1
....uggaUggagaacugauaagggcu..................................	7	1	Fm1
....uggaAggagaacugauaagggcu..................................	6	1	Fm1
....uggacggaAaacugauaagggcu..................................	2	1	Fm1
....uggacggaCaacugauaagggcu..................................	5	1	Fm1
....ugNacggagaacugauaagggcu..................................	2	1	Fm1
....uggacggCgaacugauaagggcu..................................	4	1	Fm1
....uggacggagaacugauaaggAcu..................................	5	1	Fm1
....uggacggagaacCgauaagggcu..................................	8	1	Fm1
....uCgacggagaacugauaagggcu..................................	6	1	Fm1
....uggacNgagaacugauaagggcu..................................	6	1	Fm1
....uggacggaUaacugauaagggcu..................................	5	1	Fm1
....uggacggagaacugauaaAggcu..................................	3	1	Fm1
....uggacggagaacugauaagggcu..................................	40145	0	Fm1

```
                    Star                                Mature
gaauuuguccgaaagcgaguccucgucauuuuuccgggccuguuuuuauacccuacuggacggagaacugauaagggcucgaauaggaccaagcaacauuugacguuaucugu
........................................................uggacggagaacugauaagUgcu..................................   5     1   Fm1
........................................................Cggacggagaacugauaagggcu..................................   5     1   Fm1
........................................................uggacggagaacugaAaagggcu..................................   1     1   Fm1
........................................................uggacgAagaacugauaagggcu..................................   5     1   Fm1
........................................................uggacAgagaacugauaagggcu..................................   2     1   Fm1
........................................................uggacggagaacugauaGgggcu..................................   520   1   Fm1
........................................................uggacggUgaacugauaagggcu..................................   16    1   Fm1
........................................................uUgacggagaacugauaagggcu..................................   10    1   Fm1
........................................................uggacggGgaacugauaagggcu..................................   9     1   Fm1
........................................................uggacgUagaacugauaagggcu..................................   10    1   Fm1
........................................................uggacggagaacugauCagggcu..................................   98    1   Fm1
........................................................uggacggagaacugauaaggUcu..................................   10    1   Fm1
........................................................uggacggagaacugauaagggUu..................................   885   1   Fm1
........................................................uggacggagaacugauaaUggcu..................................   6     1   Fm1
........................................................uggacggagaacugauaagCgcu..................................   3     1   Fm1
........................................................uggacgNagaacugauaagggcu..................................   3     1   Fm1
........................................................uggacggagaacugauaaggCcu..................................   4     1   Fm1
........................................................uggacggagaacugaGaagggcu..................................   1     1   Fm1
........................................................uggacggagaacuUauaagggcu..................................   5     1   Fm1
........................................................uggacggagGacugauaagggcu..................................   74    1   Fm1
........................................................uggacggagaacugauGagggcu..................................   61    1   Fm1
........................................................uggacggagaacugauaagggcG..................................   2275  1   Fm1
........................................................uggacgCagaacugauaagggcu..................................   4     1   Fm1
........................................................ugAacggagaacugauaagggcu..................................   3     1   Fm1
........................................................uggacggagaUcugauaagggcuc.................................   10    1   Fm1
........................................................uggacggagaacugauaagggUuc.................................   2     1   Fm1
........................................................uggacggagaacugauaagggAuc.................................   6     1   Fm1
........................................................uggacggagaacugauaagggcuc.................................   203   0   Fm1
........................................................uggacggagaacugauaagggcuA.................................   217   1   Fm1
........................................................uggacggagaacugauaagggcCc.................................   5     1   Fm1
........................................................uggacggagaacugauaCgggcuc.................................   1     1   Fm1
........................................................uggacggUgaacugauaagggcuc.................................   1     1   Fm1
........................................................uggacggagaacugauaagggcuG.................................   63    1   Fm1
........................................................uggacggagaacugauaagggcAc.................................   138   1   Fm1
........................................................uggacggagaacugauaagggcucA................................   1     1   Fm1
........................................................uggacggagaacugauaagggcAcg................................   1     1   Fm1
........................................................uggacggagaacugauaagggcucU................................   28    1   Fm1
........................................................uggacggagaacugauaagggcuGgaa..............................   1     1   Fm1
.........................................................ggacggaAaacugauaag......................................   1     1   Fm1
.........................................................ggacggagaacugauaaU......................................   16    1   Fm1
.........................................................ggacggagaacugauaag......................................   21    0   Fm1
.........................................................Ugacggagaacugauaag......................................   1     1   Fm1
.........................................................ggacggagaacugauaUg......................................   1     1   Fm1
.........................................................ggacggagGacugauaag......................................   1     1   Fm1
.........................................................ggGcggagaacugauaag......................................   1     1   Fm1
.........................................................ggacggagaacugauaaA......................................   8     1   Fm1
.........................................................ggacggagaacugauaagA.....................................   4     1   Fm1
.........................................................ggacggagaacugauaagU.....................................   1     1   Fm1
.........................................................ggacggagaaAugauaagg.....................................   1     1   Fm1
.........................................................Ugacggagaacugauaagg.....................................   2     1   Fm1
.........................................................ggacggagaacugauaagg.....................................   60    0   Fm1
.........................................................ggacggagaacGgauaagg.....................................   1     1   Fm1
.........................................................ggacggagCacugauaaggg....................................   1     1   Fm1
.........................................................ggacggagaacugauaaggC....................................   1     1   Fm1
.........................................................ggacCgagaacugauaaggg....................................   1     1   Fm1
.........................................................ggacggagaacugauaaggA....................................   16    1   Fm1
.........................................................ggacggagaacugauCaggg....................................   1     1   Fm1
.........................................................ggacggagaacugauGaggg....................................   1     1   Fm1
.........................................................ggacggagaaAugauaaggg....................................   1     1   Fm1
.........................................................ggacggagaacugauaaggU....................................   4     1   Fm1
.........................................................ggacggagaacugauaaggg....................................   73    0   Fm1
.........................................................Ugacggagaacugauaaggg....................................   4     1   Fm1
.........................................................ggacggaAaacugauaagggc...................................   2     1   Fm1
.........................................................ggCcggagaacugauaagggc...................................   2     1   Fm1
.........................................................ggacggagaacugauaaggCc...................................   4     1   Fm1
.........................................................ggacggGgaacugauaagggc...................................   1     1   Fm1
.........................................................ggacggagaacugauaagUgc...................................   2     1   Fm1
.........................................................ggacggagaacugauaaggAc...................................   1     1   Fm1
.........................................................gUacggagaacugauaagggc...................................   1     1   Fm1
.........................................................ggacggagaaUugauaagggc...................................   4     1   Fm1
```

Star	Mature			

gaauuuguccgaaagcgaguccucgucauuuuuccgggccuguuuuuauacccuacuggacggagaacugauaagggcucgaauaggaccaagcaacauuugacguuaucugu			
....ggacggagaacugUuaagggc...................................	1	1	Fm1
....ggacggagaacugauaaggUc...................................	2	1	Fm1
....ggacggagGacugauaagggc...................................	4	1	Fm1
....ggacggagaacugauaagggU...................................	12	1	Fm1
....ggacggagaCcugauaagggc...................................	3	1	Fm1
....ggacggCgaacugauaagggc...................................	4	1	Fm1
....ggacggagUacugauaagggc...................................	2	1	Fm1
....ggacggaUaacugauaagggc...................................	1	1	Fm1
....ggacggagCacugauaagggc...................................	16	1	Fm1
....ggacggagaacugaAaagggc...................................	2	1	Fm1
....ggacggagaaGugauaagggc...................................	1	1	Fm1
....Ugacggagaacugauaagggc...................................	45	1	Fm1
....ggacggagaacGgauaagggc...................................	1	1	Fm1
....ggaGggagaacugauaagggc...................................	1	1	Fm1
....ggacggagaacCgauaagggc...................................	3	1	Fm1
....ggGcggagaacugauaagggc...................................	2	1	Fm1
....ggacgUagaacugauaagggc...................................	1	1	Fm1
....ggaAggagaacugauaagggc...................................	4	1	Fm1
....ggacUgagaacugauaagggc...................................	1	1	Fm1
....ggacggUgaacugauaagggc...................................	1	1	Fm1
....ggacggagaacAgauaagggc...................................	1	1	Fm1
....ggacggagaacugauaGgggc...................................	9	1	Fm1
....ggacggagaacugauaagggc...................................	1101	0	Fm1
....ggacggagaacugauaUgggc...................................	1	1	Fm1
....ggacggagaacugauUagggc...................................	1	1	Fm1
....ggacggagaacugauaagAgc...................................	2	1	Fm1
....ggacggagaacugauaCgggc...................................	1	1	Fm1
....ggacggagaaAugauaagggc...................................	2	1	Fm1
....ggacggagaacugauaagggA...................................	51	1	Fm1
....ggacggagaacugauCagggc...................................	1	1	Fm1
....ggacggagaacugauaagggcG..................................	4	1	Fm1
....Ugacggagaacugauaagggcu..................................	1	1	Fm1
....ggacggagaacugauaagggAu..................................	2	1	Fm1
....ggacggagaacugauaagggcu..................................	48	0	Fm1
....ggacggagaacugauaagggcAc.................................	1	1	Fm1
....Uacggagaacugauaagggc...................................	3	1	Fm1
....gacggagaacugauaagggc...................................	4	0	Fm1
....cggagaacugauaagggc...................................	3	0	Fm1
....Uggagaacugauaagggc...................................	10	1	Fm1
...................uccucgucauuuuuccgggccu..	1	0	Mm3
....................ccucgucauuuuuccgggccu..	1	0	Mm3
....................ccucgucauuuuuccgggccugG..	2	1	Mm3
....................ccuUgucauuuuuccgggccugu..	1	1	Mm3
....................ccucgucauuuuuccggUccugu..	2	1	Mm3
....................ccucgucauuuuuccgggccugu..	31	0	Mm3
.....................cucgucauuuuuccgggccugu..	2	0	Mm3
....uuuuuauacccuacuggacggagaac...	1	0	Mm3
....uuuuauacccuacuggacggagaac...	1	0	Mm3
....uuuuauacccuacuggacggagaacugau...	2	0	Mm3
....Ucuggacggagaacugauaagggc...................................	6	1	Mm3
....cuggacggagaacugaua..	1	0	Mm3
....cuggacggagaacugauaa.......................................	3	0	Mm3
....cuggacggagaacugauaaA......................................	5	1	Mm3
....cuggacggagaacugauaag......................................	13	0	Mm3
....cuggacggagaacugauaagA.....................................	9	1	Mm3
....cuggacggagaacugauaagg.....................................	18	0	Mm3
....cuggacggagaacugauUagg.....................................	1	1	Mm3
....cuggaNggagaacugauaagg.....................................	1	1	Mm3
....cuggacggagaacugauaaggC....................................	1	1	Mm3
....cuggacggagaacugauaaggU....................................	3	1	Mm3
....cuggacggagaacugauCaggg....................................	2	1	Mm3
....cuggacggagaacugauaaggg....................................	33	0	Mm3
....cuggacggagaacugauaaggA....................................	4	1	Mm3
....cuggacggagaacugauaGggg....................................	1	1	Mm3
....cuggacggagaacugauaagggU...................................	2	1	Mm3
....cuggacggagaacugauaagggA...................................	2	1	Mm3
....cuggCcggagaacugauaagggc...................................	1	1	Mm3
....cuggacggagaacugauaagggc...................................	112	0	Mm3
....cAggacggagaacugauaagggc...................................	2	1	Mm3

```
                    Star                                  Mature
gaauuuguccgaaagcgaguccucgucauuuuuccgggccuguuuuuauacccuacuggacggagaacugauaagggcucgaauaggaccaagcaacauuugacguuaucugu
.......................................................cuggacggagaacugauaagggcu..................................   10     0     Mm3
........................................................uggacggUgaacugauaa.......................................   1      1     Mm3
........................................................uggacUgagaacugauaa.......................................   1      1     Mm3
........................................................uggacggagaacCgauaa.......................................   1      1     Mm3
........................................................uggaUggagaacugauaa.......................................   2      1     Mm3
........................................................uggacggagaacugauaU.......................................   62     1     Mm3
........................................................Nggacggagaacugauaa.......................................   2      1     Mm3
........................................................uggacggagaaUugauaa.......................................   1      1     Mm3
........................................................uggCcggagaacugauaa.......................................   1      1     Mm3
........................................................uggacggaCaacugauaa.......................................   1      1     Mm3
........................................................uggacggagaacugaAaa.......................................   66     1     Mm3
........................................................uggacggagaacugaCaa.......................................   2      1     Mm3
........................................................uggacggagaGcugauaa.......................................   3      1     Mm3
........................................................uggacggagaacugauCa.......................................   6      1     Mm3
........................................................uggacggagaCcugauaa.......................................   1      1     Mm3
........................................................uggacggagaacugauGa.......................................   2      1     Mm3
........................................................uggacggagCacugauaa.......................................   1      1     Mm3
........................................................uggacggagaacugauaa.......................................   2074   0     Mm3
........................................................Aggacggagaacugauaa.......................................   2      1     Mm3
........................................................uggacggagaacugauaC.......................................   4      1     Mm3
........................................................uggacggagaacugauUa.......................................   5      1     Mm3
........................................................uggacggagaacugauaG.......................................   13     1     Mm3
........................................................uggacggagaacCgauaag......................................   1      1     Mm3
........................................................uUgacggagaacugauaag......................................   2      1     Mm3
........................................................uggacggagaacugauUag......................................   14     1     Mm3
........................................................uggacggagaaUugauaag......................................   2      1     Mm3
........................................................uggacggagaacugauaGg......................................   40     1     Mm3
........................................................uggacggagaacugCuaag......................................   1      1     Mm3
........................................................uggacggagaGcugauaag......................................   30     1     Mm3
........................................................uggacggagaacugauaaC......................................   15     1     Mm3
........................................................uggacUgagaacugauaag......................................   2      1     Mm3
........................................................uggUcggagaacugauaag......................................   1      1     Mm3
........................................................uggacggUgaacugauaag......................................   3      1     Mm3
........................................................uggGcggagaacugauaag......................................   1      1     Mm3
........................................................uggacggagaacugauaaU......................................   1351   1     Mm3
........................................................uggacggagaacugaCaag......................................   1      1     Mm3
........................................................uggacggagaacuUauaag......................................   4      1     Mm3
........................................................Nggacggagaacugauaag......................................   3      1     Mm3
........................................................uggacggGgaacugauaag......................................   3      1     Mm3
........................................................uggacggagaaAugauaag......................................   1      1     Mm3
........................................................uggCcggagaacugauaag......................................   1      1     Mm3
........................................................uggacggagaCcugauaag......................................   14     1     Mm3
........................................................uggacggagaacugauCag......................................   11     1     Mm3
........................................................ugAacggagaacugauaag......................................   4      1     Mm3
........................................................uggacggagaacugUuaag......................................   1      1     Mm3
........................................................Aggacggagaacugauaag......................................   11     1     Mm3
........................................................uggacggagaacugauaaA......................................   5374   1     Mm3
........................................................uggacggagaacuAauaag......................................   4      1     Mm3
........................................................uggacggagaacugaAaag......................................   3      1     Mm3
........................................................uggacggagUacugauaag......................................   4      1     Mm3
........................................................uggacggagCacugauaag......................................   1      1     Mm3
........................................................uAgacggagaacugauaag......................................   3      1     Mm3
........................................................uggacAgagaacugauaag......................................   4      1     Mm3
........................................................uggacggagGacugauaag......................................   9      1     Mm3
........................................................Cggacggagaacugauaag......................................   2      1     Mm3
........................................................uggacggagaacugauGag......................................   10     1     Mm3
........................................................uggacggagaacugauaag......................................   8431   0     Mm3
........................................................uggacggaUaacugauaag......................................   2      1     Mm3
........................................................uggacggagaacugauaUg......................................   30     1     Mm3
........................................................uggacggagaacugauaCg......................................   30     1     Mm3
........................................................uggaAggagaacugauaag......................................   1      1     Mm3
........................................................uggacgUagaacugauaagg.....................................   1      1     Mm3
........................................................uggacggagaacugauaagC.....................................   10     1     Mm3
........................................................uggacggagCacugauaagg.....................................   5      1     Mm3
........................................................uggacggagaacuUauaagg.....................................   1      1     Mm3
........................................................uggacggagaacCgauaagg.....................................   1      1     Mm3
........................................................uggGcggagaacugauaagg.....................................   5      1     Mm3
........................................................Gggacggagaacugauaagg.....................................   1      1     Mm3
........................................................uggacggagaacugauaGgg.....................................   91     1     Mm3
........................................................uggacggagaacugauaagU.....................................   617    1     Mm3
```

```
                    Star                               Mature
gaauuuguccgaaagcgaguccucgucauuuuuccgggccuguuuuuauacccuacuggacggagaacugauaagggcucgaauaggaccaagcaacauuugacguuaucugu
........................................................Aggacggagaacugauaagg.....................................   140     1   Mm3
........................................................uggacggagaacugauaaUg.....................................   2       1   Mm3
........................................................uggUcggagaacugauaagg.....................................   4       1   Mm3
........................................................uggacggagaacugauaCgg.....................................   58      1   Mm3
........................................................uggacUgagaacugauaagg.....................................   11      1   Mm3
........................................................uNgacggagaacugauaagg.....................................   1       1   Mm3
........................................................uggacggagUacugauaagg.....................................   2       1   Mm3
........................................................uggacggagaacGgauaagg.....................................   2       1   Mm3
........................................................uggacCgagaacugauaagg.....................................   1       1   Mm3
........................................................uggacggagaacugauaagA.....................................   3650    1   Mm3
........................................................uggacggagGacugauaagg.....................................   27      1   Mm3
........................................................uggacggagaaAugauaagg.....................................   1       1   Mm3
........................................................ugCacggagaacugauaagg.....................................   2       1   Mm3
........................................................uggacggagaacugauGagg.....................................   21      1   Mm3
........................................................uggacggagaacugauaagg.....................................   14584   0   Mm3
........................................................uggacgCagaacugauaagg.....................................   3       1   Mm3
........................................................uggacAgagaacugauaagg.....................................   2       1   Mm3
........................................................Nggacggagaacugauaagg.....................................   6       1   Mm3
........................................................uCgacggagaacugauaagg.....................................   1       1   Mm3
........................................................uggacNgagaacugauaagg.....................................   3       1   Mm3
........................................................uggacggagaacugauUagg.....................................   12      1   Mm3
........................................................uggaUggagaacugauaagg.....................................   7       1   Mm3
........................................................uggacggagaacugauaaAg.....................................   91      1   Mm3
........................................................uggacggagaacugUuaagg.....................................   3       1   Mm3
........................................................uggacggaCaacugauaagg.....................................   1       1   Mm3
........................................................uggacggagaGcugauaagg.....................................   140     1   Mm3
........................................................uUgacggagaacugauaagg.....................................   7       1   Mm3
........................................................Cggacggagaacugauaagg.....................................   4       1   Mm3
........................................................uggacggagaacugGuaagg.....................................   1       1   Mm3
........................................................uggacggagaaUugauaagg.....................................   3       1   Mm3
........................................................uggacggaAaacugauaagg.....................................   2       1   Mm3
........................................................uggacggUgaacugauaagg.....................................   6       1   Mm3
........................................................uggacgAagaacugauaagg.....................................   2       1   Mm3
........................................................uggacggagaacugauCagg.....................................   31      1   Mm3
........................................................uggacggagaCcugauaagg.....................................   76      1   Mm3
........................................................uggacggCgaacugauaagg.....................................   2       1   Mm3
........................................................uggacggagaacuAauaagg.....................................   1       1   Mm3
........................................................uAgacggagaacugauaagg.....................................   11      1   Mm3
........................................................uggacggGgaacugauaagg.....................................   2       1   Mm3
........................................................uggacggagaacugauaUgg.....................................   64      1   Mm3
........................................................uggacggaUaacugauaagg.....................................   1       1   Mm3
........................................................ugUacggagaacugauaagg.....................................   3       1   Mm3
........................................................uggCcggagaacugauaagg.....................................   3       1   Mm3
........................................................uggacggagaacuCauaaggg....................................   2       1   Mm3
........................................................uggacggagaacugUuaaggg....................................   5       1   Mm3
........................................................uggacUgagaacugauaaggg....................................   13      1   Mm3
........................................................uggacggagCacugauaaggg....................................   5       1   Mm3
........................................................Aggacggagaacugauaaggg....................................   102     1   Mm3
........................................................uggCcggagaacugauaaggg....................................   3       1   Mm3
........................................................uggacggagaacugauUaggg....................................   38      1   Mm3
........................................................uggacggagaacugauaaAgg....................................   3       1   Mm3
........................................................uggGcggagaacugauaaggg....................................   6       1   Mm3
........................................................uggacggagaacuAauaaggg....................................   6       1   Mm3
........................................................uggacggagaacugauaUggg....................................   74      1   Mm3
........................................................uggacggagaacugauaagCg....................................   1       1   Mm3
........................................................Cggacggagaacugauaaggg....................................   11      1   Mm3
........................................................uggaAggagaacugauaaggg....................................   2       1   Mm3
........................................................uggacggagaacugCuaaggg....................................   1       1   Mm3
........................................................uggacAgagaacugauaaggg....................................   5       1   Mm3
........................................................uggaUggagaacugauaaggg....................................   3       1   Mm3
........................................................uggacggCgaacugauaaggg....................................   4       1   Mm3
........................................................uggacggagaacuUauaaggg....................................   5       1   Mm3
........................................................uggacggagaaAugauaaggg....................................   2       1   Mm3
........................................................uggacggaAaacugauaaggg....................................   1       1   Mm3
........................................................uggacggagaCcugauaaggg....................................   71      1   Mm3
........................................................uAgacggagaacugauaaggg....................................   9       1   Mm3
........................................................uggacggagaacugauaaggC....................................   89      1   Mm3
........................................................uggacggagaGcugauaaggg....................................   145     1   Mm3
........................................................uggacggGgaacugauaaggg....................................   11      1   Mm3
........................................................uggacggagaacugauaaUgg....................................   3       1   Mm3
```

Star Mature

gaauuuguccgaaagcgaguccucgucauuuuuccgggccuguuuuuauacccuacuggacggagaacugauaagggcucgaauaggaccaagcaacauuugacguuaucugu			
....uggacggagaacugauaagAg....................................	28	1	Mm3
....uggacgUagaacugauaaggg....................................	5	1	Mm3
....uggacgNagaacugauaaggg....................................	2	1	Mm3
....uggUcggagaacugauaaggg....................................	3	1	Mm3
....uggacggagaacugaAaaggg....................................	1	1	Mm3
....ugUacggagaacugauaaggg....................................	3	1	Mm3
....uggacgAagaacugauaaggg....................................	5	1	Mm3
....uggacggagaacugGuaaggg....................................	1	1	Mm3
....uggacggagaaUugauaaggg....................................	7	1	Mm3
....ugAacggagaacugauaaggg....................................	7	1	Mm3
....uCgacggagaacugauaaggg....................................	1	1	Mm3
....uggacggaUaacugauaaggg....................................	6	1	Mm3
....uggacggagaacugauaCggg....................................	118	1	Mm3
....uggacggagaacugauGaggg....................................	40	1	Mm3
....uggacggagaacugaCaaggg....................................	1	1	Mm3
....uggacggagaacugauaaggU....................................	657	1	Mm3
....uggacggUgaacugauaaggg....................................	15	1	Mm3
....uggacggaCaacugauaaggg....................................	1	1	Mm3
....Nggacggagaacugauaaggg....................................	13	1	Mm3
....uggacggagaacugauCaggg....................................	60	1	Mm3
....uggacggagaacAgauaaggg....................................	3	1	Mm3
....uggacNgagaacugauaaggg....................................	2	1	Mm3
....uggacggagGacugauaaggg....................................	35	1	Mm3
....uggacggagaacugauaagUg....................................	2	1	Mm3
....uUgacggagaacugauaaggg....................................	5	1	Mm3
....uggacCgagaacugauaaggg....................................	2	1	Mm3
....uggacggagaacugauaaggA....................................	4536	1	Mm3
....uggacggagaacCgauaaggg....................................	4	1	Mm3
....uggacggagUacugauaaggg....................................	9	1	Mm3
....Gggacggagaacugauaaggg....................................	1	1	Mm3
....uggacggagaacugauaaggg....................................	27231	0	Mm3
....uggacggagaacugauaGggg....................................	205	1	Mm3
....ugAacggagaacugauaagggc...................................	81	1	Mm3
....uggacggCgaacugauaagggc...................................	43	1	Mm3
....uggacggagaacugauaagUgc...................................	69	1	Mm3
....uggGcggagaacugauaagggc...................................	58	1	Mm3
....uggacggagaacugauaaggCc...................................	161	1	Mm3
....ugNacggagaacugauaagggc...................................	2	1	Mm3
....uggacggagCacugauaagggc...................................	54	1	Mm3
....uggCcggagaacugauaagggc...................................	70	1	Mm3
....uggacggagaacuCauaagggc...................................	19	1	Mm3
....uggacggagaacugUuaagggc...................................	56	1	Mm3
....uggaUggagaacugauaagggc...................................	74	1	Mm3
....uggacggagaaUugauaagggc...................................	74	1	Mm3
....uggacggaAaacugauaagggc...................................	19	1	Mm3
....uggaAggagaacugauaagggc...................................	29	1	Mm3
....uggacggagaacugauCagggc...................................	629	1	Mm3
....uggacAgagaacugauaagggc...................................	50	1	Mm3
....uggacggagaacugGuaagggc...................................	29	1	Mm3
....uggacggUgaacugauaagggc...................................	168	1	Mm3
....Cggacggagaacugauaagggc...................................	55	1	Mm3
....uggacggaCaacugauaagggc...................................	13	1	Mm3
....uggacggagaacuUauaagggc...................................	28	1	Mm3
....uggacggagaacugaCaagggc...................................	20	1	Mm3
....Nggacggagaacugauaagggc...................................	148	1	Mm3
....uggacggagaacugauaCgggc...................................	1119	1	Mm3
....uggacggagaacCgauaagggc...................................	35	1	Mm3
....uggacggagaacugauaagggA...................................	8184	1	Mm3
....uggacggagaacugauaagAgc...................................	49	1	Mm3
....uggacggagaacugauaaggUc...................................	74	1	Mm3
....uCgacggagaacugauaagggc...................................	32	1	Mm3
....uggacggagaacugauaagggc...................................	293785	0	Mm3
....uggUcggagaacugauaagggc...................................	42	1	Mm3
....uNgacggagaacugauaagggc...................................	3	1	Mm3
....uggacgUagaacugauaagggc...................................	53	1	Mm3
....uggacggagaacugaGaagggc...................................	2	1	Mm3
....uggacggagUacugauaagggc...................................	126	1	Mm3
....ugUacggagaacugauaagggc...................................	39	1	Mm3
....uggacggagaacugauaUgggc...................................	986	1	Mm3
....uggaGggagaacugauaagggc...................................	14	1	Mm3

```
                     Star                                  Mature
gaauuuguccgaaagcgaguccucgucauuuuuccgggccuguuuuuauacccuacuggacggagaacugauaagggcucgaauaggaccaagcaacauuugacguuaucugu
........................................................ugCacggagaacugauaagggc...................................    24    1    Mm3
........................................................uggacgNagaacugauaagggc...................................    22    1    Mm3
........................................................uggacUgagaacugauaagggc...................................    32    1    Mm3
........................................................uAgacggagaacugauaagggc...................................    111   1    Mm3
........................................................uggacggagaacugauGagggc...................................    556   1    Mm3
........................................................uggacgCagaacugauaagggc...................................    17    1    Mm3
........................................................Aggacggagaacugauaagggc...................................    284   1    Mm3
........................................................Gggacggagaacugauaagggc...................................    15    1    Mm3
........................................................uggacggagaacGgauaagggc...................................    12    1    Mm3
........................................................uggacCgagaacugauaagggc...................................    7     1    Mm3
........................................................uggacggagaCcugauaagggc...................................    247   1    Mm3
........................................................uggacNgagaacugauaagggc...................................    38    1    Mm3
........................................................uggacggagaacuAauaagggc...................................    60    1    Mm3
........................................................uggacggagaacugauaaAggc...................................    31    1    Mm3
........................................................uggacggagaaAugauaagggc...................................    9     1    Mm3
........................................................uggacggagaacugauaGgggc...................................    2855  1    Mm3
........................................................uggacggagaacugauaagggU...................................    2911  1    Mm3
........................................................uggacggagaacugauaagggG...................................    144   1    Mm3
........................................................uggacggagaacugaAaagggc...................................    10    1    Mm3
........................................................uggacggagaacugauaaUggc...................................    50    1    Mm3
........................................................uggacggagaacugauUagggc...................................    428   1    Mm3
........................................................uggacggagaacAgauaagggc...................................    17    1    Mm3
........................................................uUgacggagaacugauaagggc...................................    58    1    Mm3
........................................................uggacggagaacugauaagCgc...................................    22    1    Mm3
........................................................uggacgAagaacugauaagggc...................................    36    1    Mm3
........................................................uggacggagaacugauaNgggc...................................    2     1    Mm3
........................................................uggacggagaaGugauaagggc...................................    6     1    Mm3
........................................................uggacggagaacugCuaagggc...................................    9     1    Mm3
........................................................uggacggGgaacugauaagggc...................................    76    1    Mm3
........................................................uggacggagaacugauaaggAc...................................    60    1    Mm3
........................................................uggacggagaGcugauaagggc...................................    541   1    Mm3
........................................................uggacggagGacugauaagggc...................................    464   1    Mm3
........................................................uggacggagaacugauaaCggc...................................    7     1    Mm3
........................................................uggacggaUaacugauaagggc...................................    47    1    Mm3
........................................................uggacggagaacugauaaggUcu..................................    2     1    Mm3
........................................................uggacggagaacCgauaagggcu..................................    1     1    Mm3
........................................................uggGcggagaacugauaagggcu..................................    1     1    Mm3
........................................................uggacggGgaacugauaagggcu..................................    8     1    Mm3
........................................................uggacggagaacugauaaAggcu..................................    1     1    Mm3
........................................................uggacggagaGcugauaagggcu..................................    20    1    Mm3
........................................................uUgacggagaacugauaagggcu..................................    5     1    Mm3
........................................................uggacggagaacAgauaagggcu..................................    2     1    Mm3
........................................................uggacgUagaacugauaagggcu..................................    1     1    Mm3
........................................................uggacggagaacugUuaagggcu..................................    2     1    Mm3
........................................................uggacggagaacugauaUgggcu..................................    41    1    Mm3
........................................................uggacggagaacugauaagggcC..................................    175   1    Mm3
........................................................uggacggagaCcugauaagggcu..................................    18    1    Mm3
........................................................uggacggagaacuUauaagggcu..................................    1     1    Mm3
........................................................uggacggagaacugauCagggcu..................................    42    1    Mm3
........................................................uggacggagaacugauaagggcG..................................    434   1    Mm3
........................................................uggacggagUacugauaagggcu..................................    9     1    Mm3
........................................................uNgacggagaacugauaagggcu..................................    1     1    Mm3
........................................................uggacggagaacugauaaggCcu..................................    3     1    Mm3
........................................................uggCcggagaacugauaagggcu..................................    2     1    Mm3
........................................................uggacggagaacugGuaagggcu..................................    1     1    Mm3
........................................................uggacggagaacugauaagUgcu..................................    3     1    Mm3
........................................................uggacggagaacugauUagggcu..................................    22    1    Mm3
........................................................Cggacggagaacugauaagggcu..................................    1     1    Mm3
........................................................uggacggagaacugauaGgggcu..................................    174   1    Mm3
........................................................uggacggUgaacugauaagggcu..................................    9     1    Mm3
........................................................uggacggagaacugaCaagggcu..................................    2     1    Mm3
........................................................uggacggagGacugauaagggcu..................................    22    1    Mm3
........................................................uggacggCgaacugauaagggcu..................................    3     1    Mm3
........................................................Aggacggagaacugauaagggcu..................................    16    1    Mm3
........................................................uggacgCagaacugauaagggcu..................................    2     1    Mm3
........................................................uggacggagaacugauaagggGu..................................    3     1    Mm3
........................................................uggacgNagaacugauaagggcu..................................    1     1    Mm3
........................................................uggacggagaaUugauaagggcu..................................    4     1    Mm3
........................................................uggacggagaacugauGagggcu..................................    33    1    Mm3
........................................................uggacgAagaacugauaagggcu..................................    2     1    Mm3
```

Star **Mature**

gaauuuguccgaaagcgaguccucgucauuuuuccgggccuguuuuuauacccuacuggacggagaacugauaagggcucgaauaggaccaagcaacauuugacguuaucugu			
....uggacggagCacugauaagggcu..................................	1	1	Mm3
....uggacggagaacuCauaagggcu..................................	1	1	Mm3
....uggacggagaacugauaagggUu..................................	397	1	Mm3
....uggacggagaacugauaaUggcu..................................	2	1	Mm3
....uggacggagaacugauaagggcu..................................	14479	0	Mm3
....ugAacggagaacugauaagggcu..................................	3	1	Mm3
....uggacAgagaacugauaagggcu..................................	3	1	Mm3
....uggacNgagaacugauaagggcu..................................	3	1	Mm3
....uggacggagaacuAauaagggcu..................................	1	1	Mm3
....ugUacggagaacugauaagggcu..................................	2	1	Mm3
....ugCacggagaacugauaagggcu..................................	2	1	Mm3
....uggacggagaacugauaaggAcu..................................	3	1	Mm3
....uggacUgagaacugauaagggcu..................................	3	1	Mm3
....uggacggagaacugauaCgggcu..................................	63	1	Mm3
....uAgacggagaacugauaagggcu..................................	7	1	Mm3
....uggUcggagaacugauaagggcu..................................	1	1	Mm3
....Nggacggagaacugauaagggcu..................................	5	1	Mm3
....uggacggagaacugauaagggAu..................................	571	1	Mm3
....uggacggagaacugauaaggAcuc.................................	1	1	Mm3
....uggacggagaUcugauaagggcuc.................................	2	1	Mm3
....uggacggagaacugauaagggcuG.................................	10	1	Mm3
....uggacggagaacugauaagggcAc.................................	20	1	Mm3
....uggacggagaacugauaagggcuc.................................	34	0	Mm3
....uggacggagaacugauaagggcCc.................................	1	1	Mm3
....uggacggagaacCgauaagggcuc.................................	1	1	Mm3
....uggacggagaacugauaagggcuA.................................	44	1	Mm3
....ggacggagaacugauaaA......................................	12	1	Mm3
....ggacggagaacugauaag......................................	18	0	Mm3
....ggacggagaacugauaaU......................................	3	1	Mm3
....ggacggagaCcugauaagg.....................................	1	1	Mm3
....ggacggagaacugauaagg.....................................	33	0	Mm3
....ggacggagaacugauaagC.....................................	1	1	Mm3
....Ugacggagaacugauaagg.....................................	1	1	Mm3
....ggacggCgaacugauaagg.....................................	1	1	Mm3
....ggacggagCacugauaagg.....................................	1	1	Mm3
....ggacggagaacugauaagA.....................................	16	1	Mm3
....ggacggagaacuUauaaggg....................................	1	1	Mm3
....ggacgCagaacugauaaggg....................................	1	1	Mm3
....Ugacggagaacugauaaggg....................................	2	1	Mm3
....ggacggagCacugauaaggg....................................	2	1	Mm3
....ggacggagaacugauaaggA....................................	14	1	Mm3
....ggacggagaGcugauaaggg....................................	2	1	Mm3
....ggacggagaacugauaaggg....................................	61	0	Mm3
....ggacggagaacugauaaggU....................................	1	1	Mm3
....ggacggagaacugauaagggG...................................	1	1	Mm3
....ggacggagaacugaAaagggc...................................	1	1	Mm3
....ggacggagCacugauaagggc...................................	6	1	Mm3
....ggacggagaacugauaaggAc...................................	2	1	Mm3
....ggacggagaacugauaagUgc...................................	1	1	Mm3
....ggacggaUaacugauaagggc...................................	1	1	Mm3
....Ugacggagaacugauaagggc...................................	12	1	Mm3
....ggacggagaacugauaaggUc...................................	1	1	Mm3
....ggaAggagaacugauaagggc...................................	1	1	Mm3
....ggacggagaacugauaCgggc...................................	2	1	Mm3
....ggacggagaacugauaGgggc...................................	5	1	Mm3
....ggacggagaacugauGagggc...................................	1	1	Mm3
....ggacggagaacugauCagggc...................................	4	1	Mm3
....ggacggagaacAgauaagggc...................................	1	1	Mm3
....ggacggagaaUugauaagggc...................................	1	1	Mm3
....ggacggagaacugauaagggc...................................	621	0	Mm3
....ggacggagaacugauaagggU...................................	9	1	Mm3
....ggacCgagaacugauaagggc...................................	1	1	Mm3
....ggacggagGacugauaagggc...................................	4	1	Mm3
....ggCcggagaacugauaagggc...................................	1	1	Mm3
....ggacggagaacugauaagAgc...................................	2	1	Mm3
....ggacggagaacugauaaAggc...................................	1	1	Mm3
....ggacggaAaacugauaagggc...................................	1	1	Mm3
....ggacggagaacugauaagggA...................................	13	1	Mm3
....ggacggagaacugauaaUggc...................................	1	1	Mm3
....gUacggagaacugauaagggc...................................	2	1	Mm3

```
                   Star                            Mature
gaauuuguccgaaagcgaguccucgucauuuuuccgggccuguuuuuauacccuacuggacggagaacugauaagggcucgaauaggaccaagcaacauuugacguuaucugu
.........................................................Ngacggagaacugauaagggc...................................  1    1  Mm3
.........................................................ggacggagaaGugauaagggc...................................  1    1  Mm3
.........................................................ggacggUgaacugauaagggc...................................  1    1  Mm3
.........................................................ggacggagaCcugauaagggc...................................  1    1  Mm3
.........................................................ggacggagaacugauaUgggc...................................  1    1  Mm3
.........................................................ggacggagaaAugauaagggc...................................  1    1  Mm3
.........................................................ggacggagaacugauaaggUcu..................................  1    1  Mm3
.........................................................ggacggagaacugauaagggUu..................................  1    1  Mm3
.........................................................ggacggagaacugauaagggcu..................................  38   0  Mm3
.........................................................ggacggCgaacugauaagggcu..................................  1    1  Mm3
.........................................................ggacggagaacugauCagggcu..................................  1    1  Mm3
..........................................................gacggagaacugauaagggc...................................  3    0  Mm3
...........................................................acggagaacugauaagggc...................................  2    0  Mm3

....................ccucgucauuuuuccgggccugu......................................................................  14   0  Fm3
....................ccucgucauuuuuccgCgccugu......................................................................  1    1  Fm3
....................................................cuacuggacggagaacugauaagggc...................................  1    0  Fm3
.....................................................uacuggacggagaacugauaa.......................................  1    0  Fm3
.....................................................uacuggacggagaacugauaagggc...................................  1    0  Fm3
......................................................acuggacggagaacugaua........................................  2    0  Fm3
......................................................acuggacggagaacugauaag......................................  3    0  Fm3
......................................................acuggacggagaacugauaagg.....................................  6    0  Fm3
......................................................Ucuggacggagaacugauaagg.....................................  1    1  Fm3
......................................................acuggacggagaacugauaagU.....................................  6    1  Fm3
......................................................acuggacggagaacugauaaggA....................................  4    1  Fm3
......................................................acuggacggagaacugauaaggU....................................  7    1  Fm3
......................................................Ucuggacggagaacugauaaggg....................................  2    1  Fm3
......................................................Ucuggacggagaacugauaagggc...................................  1    1  Fm3
......................................................acuggacggagaacugauaagggc...................................  1    0  Fm3
.......................................................cuggacggagaacugaAa........................................  5    1  Fm3
.......................................................cuggacggagaacugaua........................................  12   0  Fm3
.......................................................cuggacggagaacugauU........................................  1    1  Fm3
.......................................................cuggacggagaacugauaG.......................................  1    1  Fm3
.......................................................cuggacggagaacugauaa.......................................  16   0  Fm3
.......................................................Guggacggagaacugauaag......................................  1    1  Fm3
.......................................................cuggacggagaacugauaaU......................................  11   1  Fm3
.......................................................cuggacggagaacugauaag......................................  103  0  Fm3
.......................................................cuggacggagaacugauUag......................................  1    1  Fm3
.......................................................cuggacggagaacugauaaA......................................  23   1  Fm3
.......................................................cuggacggagaacugaAaagg.....................................  1    1  Fm3
.......................................................cuggacggagaacugaCaagg.....................................  3    1  Fm3
.......................................................cuggacggagaacugauaaAg.....................................  1    1  Fm3
.......................................................cuggacggagaacugauGagg.....................................  1    1  Fm3
.......................................................cuggacggagaacugauaagA.....................................  70   1  Fm3
.......................................................cuggacggagCacugauaagg.....................................  1    1  Fm3
.......................................................cuggacggagGacugauaagg.....................................  1    1  Fm3
.......................................................cuggacggagaacugauCagg.....................................  2    1  Fm3
.......................................................cuggacggagaacugauaagU.....................................  9    1  Fm3
.......................................................cuggacggagaacugauaagg.....................................  238  0  Fm3
.......................................................cuggacggagaacugauUagg.....................................  1    1  Fm3
.......................................................cuggacggagaacugauCaggg....................................  1    1  Fm3
.......................................................Nuggacggagaacugauaaggg....................................  1    1  Fm3
.......................................................cuggacggagGacugauaaggg....................................  2    1  Fm3
.......................................................cuggacggagaacugaGaaggg....................................  1    1  Fm3
.......................................................cuggacggagaacugauaaggC....................................  3    1  Fm3
.......................................................cuggacggagaacugauaaggU....................................  46   1  Fm3
.......................................................cuggacggagaacugauaaggg....................................  284  0  Fm3
.......................................................cuggacggagaacugauGaggg....................................  3    1  Fm3
.......................................................cuggacggagaacugauaaggA....................................  67   1  Fm3
.......................................................cuggacggagCacugauaaggg....................................  2    1  Fm3
.......................................................cuggacggagaaAugauaaggg....................................  1    1  Fm3
.......................................................cuggacggagaacugauaagggU...................................  26   1  Fm3
.......................................................cuggacggGgaacugauaagggc...................................  1    1  Fm3
.......................................................cuggacggagaacugaAaagggc...................................  1    1  Fm3
.......................................................cuggacggagaacugaCaagggc...................................  1    1  Fm3
.......................................................cuggUcggagaacugauaagggc...................................  1    1  Fm3
.......................................................cuggacggagaacugauaagggA...................................  64   1  Fm3
.......................................................cuggacggagaacugauaagggc...................................  212  0  Fm3
.......................................................cuggacggagaacugauaGgggc...................................  2    1  Fm3
.......................................................cuggacggagaacugauaagggcu..................................  7    0  Fm3
```

```
                    Star                            Mature
gaauuuguccgaaagcgaguccucgucauuuuuccgggccuguuuuuauacccuacuggacggagaacugauaagggcucgaauaggaccaagcaacauuugacguuaucugu
.......................................................cuggacggagaacugaGaagggcu..................................  1      1   Fm3
........................................................uggacggagaacCgauaa.......................................  3      1   Fm3
........................................................uggacgNagaacugauaa.......................................  1      1   Fm3
........................................................uggGcggagaacugauaa.......................................  2      1   Fm3
........................................................uggacggagaacuAauaa.......................................  6      1   Fm3
........................................................uggacggagaaUugauaa.......................................  1      1   Fm3
........................................................uggacggagaacugauaa.......................................  18785  0   Fm3
........................................................uggacggagaacugaGaa.......................................  5      1   Fm3
........................................................uAgacggagaacugauaa.......................................  14     1   Fm3
........................................................uggacggagaacugauGa.......................................  23     1   Fm3
........................................................ugAacggagaacugauaa.......................................  5      1   Fm3
........................................................uggacggagaacugUuaa.......................................  5      1   Fm3
........................................................uggacgUagaacugauaa.......................................  7      1   Fm3
........................................................uggacggCgaacugauaa.......................................  1      1   Fm3
........................................................Aggacggagaacugauaa.......................................  2      1   Fm3
........................................................uggacggagCacugauaa.......................................  2      1   Fm3
........................................................uggacggaCaacugauaa.......................................  2      1   Fm3
........................................................uggacggagaacugGuaa.......................................  1      1   Fm3
........................................................Cggacggagaacugauaa.......................................  1      1   Fm3
........................................................ugUacggagaacugauaa.......................................  9      1   Fm3
........................................................uggacggagaacugaAaa.......................................  1643   1   Fm3
........................................................Nggacggagaacugauaa.......................................  6      1   Fm3
........................................................uggacggagaacugauaC.......................................  58     1   Fm3
........................................................uUgacggagaacugauaa.......................................  4      1   Fm3
........................................................uggaGggagaacugauaa.......................................  1      1   Fm3
........................................................uggUcggagaacugauaa.......................................  1      1   Fm3
........................................................uggacggagaacugauaU.......................................  591    1   Fm3
........................................................uggacggaAaacugauaa.......................................  2      1   Fm3
........................................................uggacggUgaacugauaa.......................................  7      1   Fm3
........................................................uggacUgagaacugauaa.......................................  8      1   Fm3
........................................................uggaUggagaacugauaa.......................................  6      1   Fm3
........................................................uggCcggagaacugauaa.......................................  6      1   Fm3
........................................................uggacggagaGcugauaa.......................................  27     1   Fm3
........................................................uggacggagGacugauaa.......................................  15     1   Fm3
........................................................uggacggaUaacugauaa.......................................  3      1   Fm3
........................................................uggacggagaacugauUa.......................................  54     1   Fm3
........................................................uggacggagaacugauCa.......................................  41     1   Fm3
........................................................ugCacggagaacugauaa.......................................  1      1   Fm3
........................................................uggacgAagaacugauaa.......................................  3      1   Fm3
........................................................uggacAgagaacugauaa.......................................  2      1   Fm3
........................................................uCgacggagaacugauaa.......................................  3      1   Fm3
........................................................uggacggGgaacugauaa.......................................  3      1   Fm3
........................................................uggacggagaacugauaG.......................................  105    1   Fm3
........................................................uggacggagaacGgauaa.......................................  1      1   Fm3
........................................................uggacggagaCcugauaa.......................................  13     1   Fm3
........................................................uggacggagaacuCauaa.......................................  1      1   Fm3
........................................................uggacgCagaacugauaa.......................................  1      1   Fm3
........................................................uggacggaAaacugauaag......................................  4      1   Fm3
........................................................uggaAggagaacugauaag......................................  11     1   Fm3
........................................................uggacggagaacugaAaag......................................  60     1   Fm3
........................................................ugCacggagaacugauaag......................................  8      1   Fm3
........................................................uggacggagaacugauaGg......................................  245    1   Fm3
........................................................uggacgAagaacugauaag......................................  6      1   Fm3
........................................................Aggacggagaacugauaag......................................  41     1   Fm3
........................................................uggUcggagaacugauaag......................................  14     1   Fm3
........................................................uggacggagaacugauaCg......................................  298    1   Fm3
........................................................uggacggagaacugauaag......................................  75493  0   Fm3
........................................................uggacAgagaacugauaag......................................  17     1   Fm3
........................................................uggCcggagaacugauaag......................................  11     1   Fm3
........................................................uggacggagaacugCuaag......................................  4      1   Fm3
........................................................Nggacggagaacugauaag......................................  30     1   Fm3
........................................................uggacggagaacugauCag......................................  167    1   Fm3
........................................................uggacUgagaacugauaag......................................  28     1   Fm3
........................................................uNgacggagaacugauaag......................................  2      1   Fm3
........................................................uggacgNagaacugauaag......................................  3      1   Fm3
........................................................uggaUggagaacugauaag......................................  21     1   Fm3
........................................................uCgacggagaacugauaag......................................  6      1   Fm3
........................................................uggacggagaacugauaaU......................................  15180  1   Fm3
........................................................uAgacggagaacugauaag......................................  53     1   Fm3
........................................................uggacggagaacugauaUg......................................  256    1   Fm3
```

```
                    Star                                Mature
gaauuuguccgaaagcgaguccucgucauuuuuccgggccuguuuuuauacccuacuggacggagaacugauaagggcucgaauaggaccaagcaacauuugacguuaucugu
........................................................Gggacggagaacugauaag......................................    4      1    Fm3
........................................................uggacggagaacuAauaag......................................    10     1    Fm3
........................................................uggacggagaacugaGaag......................................    2      1    Fm3
........................................................uggacggagaacugauUag......................................    116    1    Fm3
........................................................uggacggagaacugauaaC......................................    52     1    Fm3
........................................................uggacggagaGcugauaag......................................    243    1    Fm3
........................................................ugAacggagaacugauaag......................................    17     1    Fm3
........................................................uggacCgagaacugauaag......................................    2      1    Fm3
........................................................Cggacggagaacugauaag......................................    9      1    Fm3
........................................................uggacggagaacAgauaag......................................    7      1    Fm3
........................................................uggacgUagaacugauaag......................................    35     1    Fm3
........................................................uggacgCagaacugauaag......................................    7      1    Fm3
........................................................uggacggaCaacugauaag......................................    2      1    Fm3
........................................................uggacggagaacugGuaag......................................    9      1    Fm3
........................................................uggacggagaacugauGag......................................    102    1    Fm3
........................................................uggacggagCacugauaag......................................    5      1    Fm3
........................................................uggacggGgaacugauaag......................................    9      1    Fm3
........................................................uggacNgagaacugauaag......................................    7      1    Fm3
........................................................uggacggagGacugauaag......................................    72     1    Fm3
........................................................ugNacggagaacugauaag......................................    1      1    Fm3
........................................................uggacggagaaUugauaag......................................    23     1    Fm3
........................................................uggacggagaacugUuaag......................................    10     1    Fm3
........................................................uggacggagaacGgauaag......................................    1      1    Fm3
........................................................ugUacggagaacugauaag......................................    27     1    Fm3
........................................................uggacggagaCcugauaag......................................    116    1    Fm3
........................................................uggacggaUaacugauaag......................................    19     1    Fm3
........................................................uggacggagaaAugauaag......................................    2      1    Fm3
........................................................uggacggCgaacugauaag......................................    6      1    Fm3
........................................................uggaGggagaacugauaag......................................    7      1    Fm3
........................................................uUgacggagaacugauaag......................................    37     1    Fm3
........................................................uggacggagaacuUauaag......................................    8      1    Fm3
........................................................uggacggagUacugauaag......................................    18     1    Fm3
........................................................uggacggagaacugaCaag......................................    8      1    Fm3
........................................................uggacggagaacuCauaag......................................    7      1    Fm3
........................................................uggGcggagaacugauaag......................................    17     1    Fm3
........................................................uggacggagaacCgauaag......................................    9      1    Fm3
........................................................uggacggagaaGugauaag......................................    3      1    Fm3
........................................................uggacggUgaacugauaag......................................    17     1    Fm3
........................................................uggacggagaacugauaaA......................................    48211  1    Fm3
........................................................uggaGggagaacugauaagg.....................................    8      1    Fm3
........................................................uggacggaUaacugauaagg.....................................    32     1    Fm3
........................................................uggacggagaaGugauaagg.....................................    4      1    Fm3
........................................................uggacggagaacugauaaAg.....................................    936    1    Fm3
........................................................uggacggagaacugGuaagg.....................................    15     1    Fm3
........................................................Nggacggagaacugauaagg.....................................    83     1    Fm3
........................................................uggacgUagaacugauaagg.....................................    46     1    Fm3
........................................................uggacggagCacugauaagg.....................................    14     1    Fm3
........................................................Gggacggagaacugauaagg.....................................    16     1    Fm3
........................................................uggacUgagaacugauaagg.....................................    50     1    Fm3
........................................................uCgacggagaacugauaagg.....................................    18     1    Fm3
........................................................ugAacggagaacugauaagg.....................................    36     1    Fm3
........................................................Cggacggagaacugauaagg.....................................    26     1    Fm3
........................................................uggacggagaacugCuaagg.....................................    10     1    Fm3
........................................................uggacggaCaacugauaagg.....................................    11     1    Fm3
........................................................uggacggagaacugauaagC.....................................    22     1    Fm3
........................................................uggacggagaGcugauaagg.....................................    1812   1    Fm3
........................................................uggCcggagaacugauaagg.....................................    34     1    Fm3
........................................................uggacggagaacugauCagg.....................................    354    1    Fm3
........................................................uNgacggagaacugauaagg.....................................    1      1    Fm3
........................................................uggacggagaacugaCaagg.....................................    19     1    Fm3
........................................................uggacggagUacugauaagg.....................................    32     1    Fm3
........................................................ugCacggagaacugauaagg.....................................    13     1    Fm3
........................................................uggacgCagaacugauaagg.....................................    11     1    Fm3
........................................................uggacggaAaacugauaagg.....................................    10     1    Fm3
........................................................uggaAggagaacugauaagg.....................................    20     1    Fm3
........................................................uggacggagaacugauGagg.....................................    266    1    Fm3
........................................................uggacgNagaacugauaagg.....................................    9      1    Fm3
........................................................uggacgAagaacugauaagg.....................................    12     1    Fm3
........................................................uggacggagaacugauaaCg.....................................    5      1    Fm3
........................................................uggacggagaacGgauaagg.....................................    7      1    Fm3
```

```
                     Star                                   Mature
gaauuuguccgaaagcgaguccucgucauuuuuccgggccuguuuuuauacccuacuggacggagaacugauaagggcucgaauaggaccaagcaacauuugacguuaucugu
........................................................uAgacggagaacugauaagg.....................................    79       1    Fm3
........................................................uggacAgagaacugauaagg.....................................    37       1    Fm3
........................................................uggacggagaacAgauaagg.....................................    4        1    Fm3
........................................................ugUacggagaacugauaagg.....................................    42       1    Fm3
........................................................Aggacggagaacugauaagg.....................................    638      1    Fm3
........................................................uggacggagaacugauaagU.....................................    5388     1    Fm3
........................................................uggUcggagaacugauaagg.....................................    25       1    Fm3
........................................................uggacggagaacuAauaagg.....................................    33       1    Fm3
........................................................uggacggagGacugauaagg.....................................    235      1    Fm3
........................................................uggacggagaacuUauaagg.....................................    17       1    Fm3
........................................................uUgacggagaacugauaagg.....................................    57       1    Fm3
........................................................uggacggagaacugaAaagg.....................................    7        1    Fm3
........................................................uggacggagaCcugauaagg.....................................    1074     1    Fm3
........................................................uggacggGgaacugauaagg.....................................    22       1    Fm3
........................................................uggacggUgaacugauaagg.....................................    37       1    Fm3
........................................................uggacggagaacugauUagg.....................................    249      1    Fm3
........................................................uggacggagaacCgauaagg.....................................    19       1    Fm3
........................................................uggacggagaacugUuaagg.....................................    33       1    Fm3
........................................................uggacggagaaUugauaagg.....................................    31       1    Fm3
........................................................ugNacggagaacugauaagg.....................................    1        1    Fm3
........................................................uggacggagaaAugauaagg.....................................    10       1    Fm3
........................................................uggacggagaacugauaGgg.....................................    663      1    Fm3
........................................................uggacggagaacugaGaagg.....................................    6        1    Fm3
........................................................uggacggagaacugauaagg.....................................    154018   0    Fm3
........................................................uggaUggagaacugauaagg.....................................    57       1    Fm3
........................................................uggacCgagaacugauaagg.....................................    6        1    Fm3
........................................................uggacNgagaacugauaagg.....................................    17       1    Fm3
........................................................uggacggagaacugauaagA.....................................    43430    1    Fm3
........................................................uggacggagaacugauaaUg.....................................    45       1    Fm3
........................................................uggacggagaacugauaUgg.....................................    476      1    Fm3
........................................................uggacggCgaacugauaagg.....................................    9        1    Fm3
........................................................uggGcggagaacugauaagg.....................................    36       1    Fm3
........................................................uggacggagaacuCauaagg.....................................    9        1    Fm3
........................................................uggacggagaacugauaCgg.....................................    648      1    Fm3
........................................................Aggacggagaacugauaaggg....................................    389      1    Fm3
........................................................uggacgNagaacugauaaggg....................................    10       1    Fm3
........................................................uggacggagUacugauaaggg....................................    41       1    Fm3
........................................................uggacggaUaacugauaaggg....................................    41       1    Fm3
........................................................uggacggagaaUugauaaggg....................................    50       1    Fm3
........................................................uggacggagaacugauCaggg....................................    465      1    Fm3
........................................................uggacggagaaAugauaaggg....................................    6        1    Fm3
........................................................uggacggCgaacugauaaggg....................................    14       1    Fm3
........................................................uggaUggagaacugauaaggg....................................    66       1    Fm3
........................................................uggacAgagaacugauaaggg....................................    34       1    Fm3
........................................................uUgacggagaacugauaaggg....................................    58       1    Fm3
........................................................uggacggagaacugauaaggC....................................    78       1    Fm3
........................................................uggacggagGacugauaaggg....................................    195      1    Fm3
........................................................uggacggagaacAgauaaggg....................................    10       1    Fm3
........................................................Cggacggagaacugauaaggg....................................    31       1    Fm3
........................................................uggacUgagaacugauaaggg....................................    73       1    Fm3
........................................................uggacggagaacugauaaAgg....................................    22       1    Fm3
........................................................uggacggagaacuAauaaggg....................................    52       1    Fm3
........................................................uggacggagaacugauaaggA....................................    98094    1    Fm3
........................................................uggacggagaacugauaagAg....................................    189      1    Fm3
........................................................uggacggagaacugauaaUgg....................................    103      1    Fm3
........................................................uggacggagaacugauaaggg....................................    200595   0    Fm3
........................................................uggacNgagaacugauaaggg....................................    30       1    Fm3
........................................................uggacggaAaacugauaaggg....................................    13       1    Fm3
........................................................uggacggagaCcugauaaggg....................................    674      1    Fm3
........................................................uggacggagaGcugauaaggg....................................    1292     1    Fm3
........................................................uggacggagaacugUuaaggg....................................    48       1    Fm3
........................................................uggGcggagaacugauaaggg....................................    44       1    Fm3
........................................................uAgacggagaacugauaaggg....................................    98       1    Fm3
........................................................uggacggagaacuCauaaggg....................................    14       1    Fm3
........................................................ugUacggagaacugauaaggg....................................    57       1    Fm3
........................................................Nggacggagaacugauaaggg....................................    111      1    Fm3
........................................................uggacggagaacugauUaggg....................................    327      1    Fm3
........................................................uggUcggagaacugauaaggg....................................    33       1    Fm3
........................................................uggacggagaacugauaUggg....................................    709      1    Fm3
........................................................uggacggagaacugauaaggU....................................    7452     1    Fm3
```

```
                    Star                                   Mature
gaauuuguccgaaagcgaguccucgucauuuuuccgggccuguuuuuauacccuacuggacggagaacugauaagggcucgaauaggaccaagcaacauuugacguuaucugu
........................................................uggacggUgaacugauaaggg....................................    90      1     Fm3
........................................................uggacCgagaacugauaaggg....................................    7       1     Fm3
........................................................uggacggagaacugaGaaggg....................................    4       1     Fm3
........................................................uNgacggagaacugauaaggg....................................    3       1     Fm3
........................................................uggacggGgaacugauaaggg....................................    44      1     Fm3
........................................................uggacggagaacugGuaaggg....................................    33      1     Fm3
........................................................uggacggaCaacugauaaggg....................................    8       1     Fm3
........................................................uggacggagaacugauaagCg....................................    15      1     Fm3
........................................................uggaAggagaacugauaaggg....................................    20      1     Fm3
........................................................uggacggagaacugauaGggg....................................    1166    1     Fm3
........................................................ugNacggagaacugauaaggg....................................    1       1     Fm3
........................................................uggacggagCacugauaaggg....................................    17      1     Fm3
........................................................uggacggagaacugauaCggg....................................    780     1     Fm3
........................................................ugCacggagaacugauaaggg....................................    14      1     Fm3
........................................................uggacggagaacuUauaaggg....................................    25      1     Fm3
........................................................uggaGggagaacugauaaggg....................................    5       1     Fm3
........................................................uggacggagaacugauaagUg....................................    79      1     Fm3
........................................................uggacggagaacCgauaaggg....................................    22      1     Fm3
........................................................uggacgCagaacugauaaggg....................................    12      1     Fm3
........................................................uggacggagaacGgauaaggg....................................    11      1     Fm3
........................................................uggCcggagaacugauaaggg....................................    29      1     Fm3
........................................................uggacggagaacugCuaaggg....................................    11      1     Fm3
........................................................uggacggagaaGugauaaggg....................................    6       1     Fm3
........................................................uggacgAagaacugauaaggg....................................    13      1     Fm3
........................................................Gggacggagaacugauaaggg....................................    12      1     Fm3
........................................................ugAacggagaacugauaaggg....................................    49      1     Fm3
........................................................uggacgUagaacugauaaggg....................................    63      1     Fm3
........................................................uggacggagaacugauGaggg....................................    294     1     Fm3
........................................................uggacggagaacugaCaaggg....................................    20      1     Fm3
........................................................uggacggagaacugauaaCgg....................................    5       1     Fm3
........................................................uCgacggagaacugauaaggg....................................    18      1     Fm3
........................................................uggacggagaacugaAaaggg....................................    13      1     Fm3
........................................................uCgacggagaacugauaagggc...................................    85      1     Fm3
........................................................uggacggagaacugauaagCgc...................................    49      1     Fm3
........................................................uggaGggagaacugauaagggc...................................    32      1     Fm3
........................................................uggacCgagaacugauaagggc...................................    40      1     Fm3
........................................................uggacgCagaacugauaagggc...................................    46      1     Fm3
........................................................uggGcggagaacugauaagggc...................................    198     1     Fm3
........................................................uggacggagaacuCauaagggc...................................    58      1     Fm3
........................................................uggacgNagaacugauaagggc...................................    41      1     Fm3
........................................................uAgacggagaacugauaagggc...................................    387     1     Fm3
........................................................uggacggagaaUugauaagggc...................................    189     1     Fm3
........................................................uggacggCgaacugauaagggc...................................    42      1     Fm3
........................................................uggacggGgaacugauaagggc...................................    180     1     Fm3
........................................................uggacggagaacGgauaagggc...................................    25      1     Fm3
........................................................uggacggagaacAgauaagggc...................................    51      1     Fm3
........................................................uggacggagaacugauaGgggc...................................    5830    1     Fm3
........................................................uggCcggagaacugauaagggc...................................    161     1     Fm3
........................................................uggacggagaacugauaagUgc...................................    281     1     Fm3
........................................................uggacggagaGcugauaagggc...................................    1460    1     Fm3
........................................................Cggacggagaacugauaagggc...................................    141     1     Fm3
........................................................ugCacggagaacugauaagggc...................................    61      1     Fm3
........................................................uggacggagaacugauNagggc...................................    3       1     Fm3
........................................................uggacggagUacugauaagggc...................................    123     1     Fm3
........................................................uggacggagaacugauaaUggc...................................    308     1     Fm3
........................................................uggacggagaaGugauaagggc...................................    15      1     Fm3
........................................................uggacggUgaacugauaagggc...................................    214     1     Fm3
........................................................uggacgAagaacugauaagggc...................................    78      1     Fm3
........................................................uggacAgagaacugauaagggc...................................    155     1     Fm3
........................................................uggaAggagaacugauaagggc...................................    91      1     Fm3
........................................................uggacggagaacugauaagAgc...................................    104     1     Fm3
........................................................uggacggagaacugauaaNggc...................................    1       1     Fm3
........................................................ugAacggagaacugauaagggc...................................    192     1     Fm3
........................................................uggacggagaacugauaaAggc...................................    51      1     Fm3
........................................................uNgacggagaacugauaagggc...................................    10      1     Fm3
........................................................uggacggagaacugaCaagggc...................................    82      1     Fm3
........................................................ugUacggagaacugauaagggc...................................    244     1     Fm3
........................................................uggacggagaacuUauaagggc...................................    83      1     Fm3
........................................................uggacUgagaacugauaagggc...................................    245     1     Fm3
........................................................uggacggagaacugauUagggc...................................    911     1     Fm3
```

```
                 Star                             Mature
gaauuuguccgaaagcgaguccucgucauuuuuccgggccuguuuuuauacccuacuggacggagaacugauaagggcucgaauaggaccaagcaacauuugacguuaucugu
........................................................uggacNgagaacugauaagggc...................................  104     1  Fm3
........................................................uggacggagaacugGuaagggc...................................  95      1  Fm3
........................................................uggacggagaacugauaaggUc...................................  273     1  Fm3
........................................................uggacggagaacugauaaCggc...................................  38      1  Fm3
........................................................uggacgUagaacugauaagggc...................................  263     1  Fm3
........................................................uggacggagaacugauaagggU...................................  12581   1  Fm3
........................................................uggacggagaacugauaagggG...................................  97      1  Fm3
........................................................uggacggagCacugauaagggc...................................  55      1  Fm3
........................................................uggacggagaaAugauaagggc...................................  34      1  Fm3
........................................................uggacggagaacugauaUgggc...................................  2622    1  Fm3
........................................................uggacggaUaacugauaagggc...................................  182     1  Fm3
........................................................uggacggagaacuAauaagggc...................................  167     1  Fm3
........................................................uggUcggagaacugauaagggc...................................  147     1  Fm3
........................................................uggacggagaCcugauaagggc...................................  696     1  Fm3
........................................................uggacggagaacugauCagggc...................................  1554    1  Fm3
........................................................uggacggagaacCgauaagggc...................................  90      1  Fm3
........................................................uggacggagaacugauaaggAc...................................  157     1  Fm3
........................................................Nggacggagaacugauaagggc...................................  405     1  Fm3
........................................................uggacggagaacugauaagggc...................................  790111  0  Fm3
........................................................uggacggagaacugaAaagggc...................................  29      1  Fm3
........................................................uggacggaCaacugauaagggc...................................  45      1  Fm3
........................................................uggacggaAaacugauaagggc...................................  51      1  Fm3
........................................................uggaUggagaacugauaagggc...................................  278     1  Fm3
........................................................uUgacggagaacugauaagggc...................................  256     1  Fm3
........................................................uggacggagaacugauGagggc...................................  1829    1  Fm3
........................................................uggacggagaacugauaaggCc...................................  71      1  Fm3
........................................................uggacggagaacugauaCgggc...................................  3025    1  Fm3
........................................................uggacggagGacugauaagggc...................................  581     1  Fm3
........................................................uggacggagaacugaGaagggc...................................  19      1  Fm3
........................................................uggacggagaacugUuaagggc...................................  135     1  Fm3
........................................................uggacggagaacugCuaagggc...................................  28      1  Fm3
........................................................Gggacggagaacugauaagggc...................................  40      1  Fm3
........................................................uggacggagaacugauaagggA...................................  100006  1  Fm3
........................................................ugNacggagaacugauaagggc...................................  1       1  Fm3
........................................................Aggacggagaacugauaagggc...................................  237     1  Fm3
........................................................uggacggagaacugCuaagggcu..................................  1       1  Fm3
........................................................uggacggagaacugauaagCgcu..................................  1       1  Fm3
........................................................uggacggagaacugauaaUggcu..................................  6       1  Fm3
........................................................uAgacggagaacugauaagggcu..................................  12      1  Fm3
........................................................Nggacggagaacugauaagggcu..................................  10      1  Fm3
........................................................uggGcggagaacugauaagggcu..................................  4       1  Fm3
........................................................uggacggagaacAgauaagggcu..................................  1       1  Fm3
........................................................uggacggagaacugauaagUgcu..................................  4       1  Fm3
........................................................uggacggagaacuUauaagggcu..................................  1       1  Fm3
........................................................uggacggagaaGugauaagggcu..................................  1       1  Fm3
........................................................uggacggagaGcugauaagggcu..................................  35      1  Fm3
........................................................uggacggagaacugauaagggGu..................................  12      1  Fm3
........................................................uggacggagaacCgauaagggcu..................................  3       1  Fm3
........................................................uggacggagaacugaAaagggcu..................................  1       1  Fm3
........................................................uggacggagaacugauaagggAu..................................  5158    1  Fm3
........................................................uggacggGgaacugauaagggcu..................................  5       1  Fm3
........................................................uggacggagCacugauaagggcu..................................  2       1  Fm3
........................................................uggacggUgaacugauaagggcu..................................  5       1  Fm3
........................................................uggacggagaacuCauaagggcu..................................  1       1  Fm3
........................................................uggacggagaacugGuaagggcu..................................  3       1  Fm3
........................................................uggacggagaacugauaagggcu..................................  17693   0  Fm3
........................................................uggacggagaaUugauaagggcu..................................  10      1  Fm3
........................................................uUgacggagaacugauaagggcu..................................  6       1  Fm3
........................................................uggacggagaacugauaGgggcu..................................  110     1  Fm3
........................................................uggacggagaacugauaCgggcu..................................  73      1  Fm3
........................................................uCgacggagaacugauaagggcu..................................  2       1  Fm3
........................................................Aggacggagaacugauaagggcu..................................  2       1  Fm3
........................................................uggacggCgaacugauaagggcu..................................  1       1  Fm3
........................................................uggacNgagaacugauaagggcu..................................  3       1  Fm3
........................................................uggacggagaacugauaagggcC..................................  86      1  Fm3
........................................................uggacggaCaacugauaagggcu..................................  1       1  Fm3
........................................................uggacggagGacugauaagggcu..................................  20      1  Fm3
........................................................uggacggagaacugauaaggAcu..................................  3       1  Fm3
........................................................uggacggagaacugauaUgggcu..................................  46      1  Fm3
........................................................uggacggagaacugauaagggUu..................................  1283    1  Fm3
```

Star **Mature**

gaauuuguccgaaagcgaguccucgucauuuuuccgggccuguuuuuauacccuacuggacggagaacugauaagggcucgaauaggaccaagcaacauuugacguuaucugu			
....uggacUgagaacugauaagggcu..................................	1	1	Fm3
....uggacggagaacugauCagggcu..................................	32	1	Fm3
....uggacggagaacugUuaagggcu..................................	3	1	Fm3
....Cggacggagaacugauaagggcu..................................	1	1	Fm3
....ugUacggagaacugauaagggcu..................................	6	1	Fm3
....uggacggagaaAugauaagggcu..................................	1	1	Fm3
....uggacggagaacuAauaagggcu..................................	3	1	Fm3
....uggacggagaacugauaaAggcu..................................	3	1	Fm3
....uggUcggagaacugauaagggcu..................................	6	1	Fm3
....uggaAggagaacugauaagggcu..................................	1	1	Fm3
....uggaUggagaacugauaagggcu..................................	9	1	Fm3
....uggacggagaacugauaagAgcu..................................	2	1	Fm3
....uggacggagaacugauUagggcu..................................	24	1	Fm3
....uggacggaUaacugauaagggcu..................................	4	1	Fm3
....uggacggagaCcugauaagggcu..................................	15	1	Fm3
....ugAacggagaacugauaagggcu..................................	5	1	Fm3
....uggCcggagaacugauaagggcu..................................	5	1	Fm3
....uggacggagUacugauaagggcu..................................	4	1	Fm3
....uggacggagaacugauaaggCcu..................................	2	1	Fm3
....uggacggagaacugauaaggUcu..................................	7	1	Fm3
....uggacggagaacugauGagggcu..................................	34	1	Fm3
....uggacggagaacugauaagggcG..................................	246	1	Fm3
....uggacAgagaacugauaagggcu..................................	6	1	Fm3
....uggacgUagaacugauaagggcu..................................	7	1	Fm3
....uggacggagaacugauaagggcAc.................................	9	1	Fm3
....uggacggagaacugauaagggcuG.................................	3	1	Fm3
....uggacggagaacugauaagggcuA.................................	60	1	Fm3
....uggacggagaacugauaagggcuc.................................	4	0	Fm3
....ggacggagaacugaGaag......................................	1	1	Fm3
....ggacggagaacugauaCg......................................	1	1	Fm3
....ggacggagaacugauaaA......................................	68	1	Fm3
....ggacggagaacugaAaag......................................	1	1	Fm3
....ggacggagaacugauCag......................................	1	1	Fm3
....ggacggagaacugauaag......................................	135	0	Fm3
....ggacggagCacugauaag......................................	1	1	Fm3
....ggaUggagaacugauaag......................................	1	1	Fm3
....ggGcggagaacugauaag......................................	1	1	Fm3
....ggacggagaacugauaaC......................................	1	1	Fm3
....ggacggaAaacugauaag......................................	1	1	Fm3
....Ugacggagaacugauaag......................................	6	1	Fm3
....ggacggagaacugauaaU......................................	21	1	Fm3
....Ugacggagaacugauaagg.....................................	9	1	Fm3
....ggacggagaaAugauaagg.....................................	3	1	Fm3
....ggacggagaacGgauaagg.....................................	1	1	Fm3
....ggacggCgaacugauaagg.....................................	1	1	Fm3
....ggacggagaacugaAaagg.....................................	1	1	Fm3
....ggacggagaacCgauaagg.....................................	2	1	Fm3
....ggacggaCaacugauaagg.....................................	1	1	Fm3
....ggacggagaacugauCagg.....................................	3	1	Fm3
....Ngacggagaacugauaagg.....................................	1	1	Fm3
....ggGcggagaacugauaagg.....................................	1	1	Fm3
....ggacggagaacugauaGgg.....................................	2	1	Fm3
....ggacggagaacugauaUgg.....................................	1	1	Fm3
....ggacggagaacugauaaUg.....................................	2	1	Fm3
....ggaAggagaacugauaagg.....................................	1	1	Fm3
....ggacggagaacugauaaAg.....................................	1	1	Fm3
....ggacggaAaacugauaagg.....................................	1	1	Fm3
....ggacggagaGcugauaagg.....................................	6	1	Fm3
....ggacggagaacugauaagA.....................................	108	1	Fm3
....ggacggagaaUugauaagg.....................................	1	1	Fm3
....ggacggagaCcugauaagg.....................................	7	1	Fm3
....ggacggagaacugauaagg.....................................	317	0	Fm3
....ggacggagaacugauaagU.....................................	8	1	Fm3
....ggacggagaacugGuaaggg....................................	1	1	Fm3
....ggacggagaacugaAaaggg....................................	1	1	Fm3
....ggacggagaGcugauaaggg....................................	4	1	Fm3
....ggacggagaaGugauaaggg....................................	2	1	Fm3
....ggacggUgaacugauaaggg....................................	1	1	Fm3
....ggacggagCacugauaaggg....................................	1	1	Fm3
....ggacggagaacugauUaggg....................................	2	1	Fm3

```
                                        Star                      Mature
gaauuuguccgaaagcgaguccucgucauuuuuccgggccuguuuuuauacccuacuggacggagaacugauaagggcucgaauaggaccaagcaacauuugacguuaucugu
.........................................................ggacggagaacugauaCggg....................................	3	1	Fm3
.........................................................ggacggagaacugauCaggg....................................	5	1	Fm3
.........................................................ggacggagaacugauaGggg....................................	3	1	Fm3
.........................................................ggacggagaacugauaaggg....................................	386	0	Fm3
.........................................................Ugacggagaacugauaaggg....................................	14	1	Fm3
.........................................................ggacggagaacGgauaaggg....................................	1	1	Fm3
.........................................................ggacggagaacugauaaggC....................................	5	1	Fm3
.........................................................ggacggagaacugauaaggA....................................	253	1	Fm3
.........................................................ggacggagaacCgauaaggg....................................	2	1	Fm3
.........................................................ggacggagaacugauaaggU....................................	16	1	Fm3
.........................................................ggacggagaCcugauaaggg....................................	5	1	Fm3
.........................................................ggacgAagaacugauaaggg....................................	1	1	Fm3
.........................................................ggacggagaacugUuaaggg....................................	1	1	Fm3
.........................................................ggacggagUacugauaaggg....................................	1	1	Fm3
.........................................................ggacggagaacugauaaAgg....................................	1	1	Fm3
.........................................................ggacggagaaAugauaaggg....................................	1	1	Fm3
.........................................................ggacggagaaUugauaagggc...................................	3	1	Fm3
.........................................................ggacggagaacugauaaggUc...................................	6	1	Fm3
.........................................................ggacggagCacugauaagggc...................................	15	1	Fm3
.........................................................ggacggagaCcugauaagggc...................................	4	1	Fm3
.........................................................ggaGggagaacugauaagggc...................................	1	1	Fm3
.........................................................ggacggagaacugauaaAggc...................................	2	1	Fm3
.........................................................ggacggagaacugaGaagggc...................................	2	1	Fm3
.........................................................ggacggagaacAgauaagggc...................................	1	1	Fm3
.........................................................ggacggagaacugauaCgggc...................................	3	1	Fm3
.........................................................ggaAggagaacugauaagggc...................................	4	1	Fm3
.........................................................ggacggagaacugauaagggc...................................	1615	0	Fm3
.........................................................ggGcggagaacugauaagggc...................................	3	1	Fm3
.........................................................ggacggagaGcugauaagggc...................................	3	1	Fm3
.........................................................ggacggagaaGugauaagggc...................................	4	1	Fm3
.........................................................ggacggagaacuCauaagggc...................................	1	1	Fm3
.........................................................Ugacggagaacugauaagggc...................................	34	1	Fm3
.........................................................ggacggagaacugauaUgggc...................................	1	1	Fm3
.........................................................Ngacggagaacugauaagggc...................................	1	1	Fm3
.........................................................ggacggagaacugauGagggc...................................	2	1	Fm3
.........................................................ggacggagaacugauaaUggc...................................	1	1	Fm3
.........................................................ggacggagaacuUauaagggc...................................	2	1	Fm3
.........................................................ggCcggagaacugauaagggc...................................	1	1	Fm3
.........................................................ggacggagaacugaAaagggc...................................	1	1	Fm3
.........................................................ggacggCgaacugauaagggc...................................	6	1	Fm3
.........................................................ggacggagaacugauaagggU...................................	23	1	Fm3
.........................................................ggacggagaacugauaaggAc...................................	8	1	Fm3
.........................................................ggacCgagaacugauaagggc...................................	3	1	Fm3
.........................................................ggacggagaacugUuaagggc...................................	1	1	Fm3
.........................................................ggacggagUacugauaagggc...................................	2	1	Fm3
.........................................................ggacggagGacugauaagggc...................................	1	1	Fm3
.........................................................ggacggagaacugauaagCgc...................................	1	1	Fm3
.........................................................ggacggaCaacugauaagggc...................................	1	1	Fm3
.........................................................ggacggUgaacugauaagggc...................................	1	1	Fm3
.........................................................ggacggaAaacugauaagggc...................................	2	1	Fm3
.........................................................ggacggagaacCgauaagggc...................................	1	1	Fm3
.........................................................ggacggagaacugauaGgggc...................................	7	1	Fm3
.........................................................ggacggagaacugauaagAgc...................................	3	1	Fm3
.........................................................ggacggagaacugauaagggA...................................	230	1	Fm3
.........................................................ggacggagaacugauaaggCc...................................	7	1	Fm3
.........................................................ggacggagaaAugauaagggc...................................	7	1	Fm3
.........................................................ggacggagaacugauUagggc...................................	2	1	Fm3
.........................................................ggacggagaacGgauaagggc...................................	6	1	Fm3
.........................................................ggacggagaacugauaagggUu..................................	3	1	Fm3
.........................................................ggacggagaacugauaagggcu..................................	26	0	Fm3
.........................................................Ugacggagaacugauaagggcu..................................	2	1	Fm3
.........................................................ggacggagaacugauaagggAu..................................	10	1	Fm3
.........................................................ggacggagaacugauaagggcAc.................................	1	1	Fm3
..........................................................gacggagaacugauaaggg....................................	3	0	Fm3
..........................................................Uacggagaacugauaaggg....................................	3	1	Fm3
..........................................................gacggagaacugauaagggA...................................	1	1	Fm3
..........................................................gacggagaacugauaagggc...................................	9	0	Fm3
..........................................................gacggagaaUugauaagggc...................................	2	1	Fm3
..........................................................Uacggagaacugauaagggc...................................	6	1	Fm3
...........................................................acggagaacugauaagggc...................................	1	0	Fm3
```

```
                    Star                                   Mature
gaauuuguccgaaagcgaguccucgucauuuuuccgggccuguuuuuauacccuacuggacggagaacugauaagggcucgaauaggaccaagcaacauuugacguuaucugu
...........................................................acggagaacugauaagggcu..................................    1    0    Fm3
............................................................Uggagaacugauaagggc...................................    10   1    Fm3
............................................................cggagaacugauaagggc...................................    1    0    Fm3
............................................................Uggagaacugauaagggcu..................................    1    1    Fm3
```
